# Minimally Invasive Interventions for Childhood Caries: A Scoping Review of Their Applicability in Public Health and Community Settings

**DOI:** 10.3390/healthcare14091155

**Published:** 2026-04-25

**Authors:** Giovanna Lima Fortunato, Gabriel Pereira Nunes, Isabela dos Santos de Deus, Priscila Toninatto Alves de Toledo, Guilherme Assumpção Silva, Cristina Antoniali Silva, Aimée Maria Guiotti, Daniela Atili Brandini

**Affiliations:** 1 Department of Dental Materials and Prosthodontics, Araçatuba School of Dentistry, São Paulo State University (UNESP), Araçatuba 16015-050, SP, Brazil; giovanna.l.fortunato@unesp.br (G.L.F.); isabela.s.deus@unesp.br (I.d.S.d.D.); aimee.m.guiotti@unesp.br (A.M.G.); 2 Department of Prosthodontics and Periodontology, Piracicaba Dental School, State University of Campinas (UNICAMP), Piracicaba 13414-903, SP, Brazil; gabriel.nunesp94@gmail.com; 3 Department of Operative Dentistry, Endodontics and Dental Materials, Bauru School of Dentistry, University of São Paulo (USP), Bauru 17012-901, SP, Brazil; priscilatoninatto@hotmail.com; 4 Department of Diagnosis and Surgery, School of Dentistry, São Paulo State University (UNESP), Araçatuba 16015-050, SP, Brazil; guilherme.assumpcao@unesp.br; 5 Department of Basic Sciences, School of Dentistry, São Paulo State University (UNESP), Araçatuba 16015-050, SP, Brazil; cristina.antoniali@unesp.br

**Keywords:** dental caries, minimally invasive dentistry, pediatric dentistry, bioactive materials, atraumatic restorative treatment

## Abstract

**Background/Objectives:** Dental caries is one of the most prevalent chronic diseases in childhood, disproportionately affecting socially vulnerable populations. This scoping review aimed to analyze the clinical effects of selected minimally invasive materials and approaches, specifically mouthrinses, fluoride varnishes, silver diamine fluoride, and glass ionomer-based interventions, for the prevention and management of dental caries in pediatric patients, with emphasis on public health and community-based settings. **Methods:** This scoping review followed the Population, Concept, and Context (PCC) framework. Electronic searches were conducted up to 23 January 2026, using tailored strategies for mouthrinses, fluoride varnishes, silver diamine fluoride (SDF), and glass ionomer cements (GICs). Randomized clinical trials (RCTs) were included. Data extraction and qualitative synthesis focused on clinical outcomes and applicability in public health contexts. **Results:** Fifty-five RCTs were included. Fluoride- or chlorhexidine-based mouthrinses showed potential in controlling cariogenic biofilm, with evidence primarily based on microbiological outcomes. Fluoride varnishes were associated with enamel remineralization and control of early white spot lesions, particularly in supervised programs. SDF was reported to achieve high caries’ arrest rates in cavitated dentin lesions of primary teeth, while its preventive effect on sound surfaces appeared comparable to other fluoride-based interventions. GICs were associated with acceptable clinical performance as pit-and-fissure sealants and in atraumatic restorative treatment. **Conclusions:** Minimally invasive dentistry (MID) approaches show promise for the prevention and management of childhood dental caries in public health and community-based settings. However, these findings should be interpreted with caution due to the heterogeneity of interventions and outcome measures, the predominance of short-term and surrogate (microbiological) outcomes, and the absence of a formal risk-of-bias assessment. As a scoping review, the synthesis is narrative in nature, which limits the ability to draw definitive conclusions. Further studies with standardized clinical outcomes and longer follow-up are needed to strengthen the evidence.

## 1. Introduction

Dental caries is chronic, multifactorial, and dynamic disease resulting from an imbalance between the demineralization and remineralization processes of dental tissues [1]. Its development is influenced by the interaction between cariogenic biofilm, frequent sugar consumption, and host-related factors. For this reason, it is widely recognized as one of the most prevalent chronic diseases in childhood [2,3]. In early childhood, this process is referred to as early childhood caries (ECC), characterized by the presence of cavitated or non-cavitated lesions, restored or missing teeth, in children under six years of age, representing a significant public health problem on a global scale [4,5]. The etiology of ECC is complex and involves the interaction of biological behavioral and social factors. Among these, early colonization by cariogenic microorganisms, inadequate oral hygiene practices, sugar-rich dietary habits, and inequalities in access to healthcare services are particularly relevant [6]. Early onset of the disease is associated with pain, infections, and impairments in growth, development, and quality of life. In addition, it increases the risk of caries recurrence throughout life [7].

Due to the high prevalence of dental caries and the population’s limited access to conventional dental treatments, minimally invasive dentistry (MID) has been established as a contemporary philosophy of oral healthcare [8]. This approach emphasizes early disease detection, understanding of the etiological factors involved, and interruption of its progression, resorting to restorative treatment only when necessary [9]. The main objective of MID is to preserve healthy dental structure as much as possible, prioritizing prevention strategies, remineralization, and minimally invasive restorative interventions [10,11].

In this context, public health actions aimed at preventing dental caries in childhood are based on an integrated approach that combines health promotion, early preventive interventions, and the reduction in inequalities in access to dental services [12]. These strategies emphasize the inclusion of oral health in primary care and the school environment [13]. In this way, school-based dental monitoring programs play a strategic role, as they allow systematic interventions during a critical period of dental development, and promote the consolidation of healthy habits from the earliest years of life [13]. Furthermore, health education directed at parents, caregivers, and students, combined with intersectoral collaboration between health, education, and social assistance, enhances the effectiveness of these actions, and contributes to the promotion of equity in children’s oral health, notably in socially vulnerable populations [5].

The contemporary management of ECC has incorporated minimally invasive preventive, and therapeutic strategies consistent with the MID philosophy and broadly applicable in settings outside the traditional dental office. Interventions such as antimicrobial solutions, topical fluoride applications, and bioactive restorative materials have been associated with reductions in cariogenic bacterial load and improved biofilm control. These effects may contribute to the prevention or arrest of caries lesions in children in school and community settings; however, this relationship is complex and influenced by multiple biological, behavioral, and environmental factors, and should not be interpreted as a direct indicator of clinical effectiveness. In this context, materials such as silver diamine fluoride (SDF) and glass ionomer cements (GICs), combined with atraumatic restorative techniques, have demonstrated favorable clinical outcomes, particularly due to their antibacterial and remineralizing properties and their feasibility in settings with limited infrastructure [14,15].

Given the diversity of available materials and approaches, it is essential to critically evaluate existing clinical evidence. Although minimally invasive dentistry encompasses a wide range of preventive and therapeutic strategies, the present review focuses on selected interventions with greater applicability in settings outside the traditional dental office, namely mouthrinses, fluoride varnishes, silver diamine fluoride, and glass ionomer–based approaches. These intervention categories were selected based on their clinical relevance within minimally invasive dentistry, their feasibility and relevance in public health and community-based settings, and their potential for implementation in contexts with limited infrastructure. Therefore, this scoping review aims to map and analyze the clinical effects of these minimally invasive interventions in pediatric populations, with a particular focus on their applicability within settings outside the traditional dental office and community-based settings.

## 2. Materials and Methods

This scoping review was conducted in accordance with the Preferred Reporting Items for Systematic Reviews and Meta-Analyses extension for Scoping Reviews (PRISMA-ScR) guidelines [16,17] and was informed by methodological approaches described in recent publications [18,19]. This scoping review was registered in Open Science Framework (Registration DOI 10.17605/OSF.IO/JR254).

The review was structured according to the Population, Concept, and Context (PCC) framework, as recommended for scoping reviews [20].

The aim of this scoping review was to map and analyze the clinical effects of selected minimally invasive materials and approaches, specifically mouthrinses, fluoride varnishes, silver diamine fluoride, and glass ionomer–based interventions used in minimally invasive dentistry for the prevention and management of dental caries in pediatric populations, with emphasis on outside the traditional dental office and community-based settings.

The guiding research question was formulated as follows: What are the clinical effects of selected minimally invasive materials and approaches, specifically mouthrinses, fluoride varnishes, silver diamine fluoride and glass ionomer based interventions, in the prevention and treatment of dental caries in childhood, and how applicable are these interventions in public health contexts?

The PCC elements were defined as follows:

- Population: Studies involving children and adolescents, including primary and mixed dentition, as well as permanent teeth in eruption, evaluated in clinical or community-based settings. Studies conducted in school, daycare, or primary health care environments were also considered eligible.
- Concept: Clinical use of selected minimally invasive materials and approaches aimed at preventing, controlling, arresting, or treating dental caries, including fluoride-based interventions (such as varnishes and mouthrinses), silver diamine fluoride and glass ionomer–based materials applied in pediatric dentistry.
- Context: Outside-office interventions in pediatric dental care settings, with outcomes related to caries prevention, lesion arrest, progression control, restorative success, or associated clinical and microbiological parameters.

### 2.1. Eligibility Criteria

The inclusion criteria encompassed randomized clinical trials (RCTs) that investigated materials and approaches used in minimally invasive dentistry for the prevention or treatment of dental caries in childhood, including interventions performed in non-clinical or non-ambulatory settings. Eligible studies involved pediatric populations (children and/or adolescents) and evaluated minimally invasive materials, such as antimicrobial agents, fluoride-based products, bioactive restorative materials or atraumatic techniques, reporting clinical outcomes related to caries prevention, control, arrest or restorative management. No restrictions were applied regarding publication year or language to comprehensively capture the available body of evidence and minimize potential selection bias. The inclusion of randomized clinical trials (RCTs) was adopted to ensure greater methodological rigor and a higher level of clinical evidence regarding the effectiveness of minimally invasive interventions in the management of dental caries in pediatric populations. This approach is further justified by the need to evaluate interventions often implemented outside the traditional dental office, in real-world health promotion settings, where RCTs allow for the estimation of clinical effects under controlled conditions. The exclusion criteria comprised non-clinical studies as well as secondary studies such as narrative reviews, systematic reviews and case reports. Studies that did not address dental caries in pediatric populations or that failed to evaluate preventive or therapeutic interventions relevant to minimally invasive dentistry were also excluded. In addition, studies without an appropriate comparator or control group when required by the study design were not considered eligible. The inclusion of randomized clinical trials was intentional, aiming to ensure a minimum level of methodological rigor and to focus on evidence reporting clinical effects under controlled conditions, while still allowing a broad mapping of interventions relevant to public health contexts.

### 2.2. Information Sources and Search Strategy

Searches were conducted in the PubMed/MEDLINE, Web of Science and Embase databases. The search strategies were adapted to each database and performed separately for each material evaluated in this review. Independent and specifically tailored strategies were developed to identify studies addressing the use of preventive or therapeutic materials in the management of dental caries in childhood. The complete search strategies used for each material are presented in Supplementary Table S1. The search strategy was intentionally designed to prioritize sensitivity, combining broad caries-related terms with intervention-specific keywords to ensure comprehensive retrieval of relevant studies. Conceptually related interventions were grouped within broader search blocks based on their functional similarity and relevance to minimally invasive approaches in pediatric dentistry rather than strict equivalence in formulation or mechanism. This approach is consistent with a scoping review methodology which aims to capture the breadth of available evidence. The broad retrieval was subsequently refined through clearly defined eligibility criteria to maintain conceptual relevance. The search included studies published up to 23 January 2026. All retrieved references were imported into reference management software (Zotero, version 6.0; Corporation for Digital Scholarship, Vienna, VA, USA) for organization and duplicate removal. In addition, a manual search of the reference lists of the included studies was performed to identify further relevant articles, ensuring comprehensive coverage of the available evidence. These intervention categories were selected based on their clinical relevance within minimally invasive dentistry, their applicability and feasibility in public health and community-based settings and their potential for implementation in contexts with limited infrastructure.

### 2.3. Study Selection

The study selection process was conducted in two stages. In the first stage, titles and abstracts were independently screened by two reviewers (GF and ISD) to identify potentially eligible studies. In the second stage, full-text versions of the selected articles were assessed for eligibility according to the predefined criteria. Any disagreements between the two reviewers were resolved through discussion, and when consensus could not be reached, a third reviewer (GPN) was consulted. In addition, a manual search of the reference lists of the included studies was performed to identify further relevant articles and the reasons for exclusion at the full-text stage were recorded and presented in the flow diagram. Agreement between the two reviewers during title and abstract screening was assessed using Cohen’s kappa coefficient (κ).

### 2.4. Data Extraction and Synthesis

Data were independently extracted by two reviewers using a standardized and previously tested data collection form to ensure consistency and reliability. The extracted information included author and year of publication, country, population characteristics, interventions, protocol, assessment method, follow-up, and main results. Data charting was performed in duplicate, and any discrepancies were resolved through discussion between the reviewers. No contact with study authors was performed to obtain or confirm additional data. The synthesis of results was conducted qualitatively, focusing on clinical outcomes related to caries prevention, control, arrest, and restorative management, as well as on the feasibility and applicability of the interventions in public health and community-based settings. The synthesis was further structured according to outcome categories, including caries’ arrest, caries’ incidence, microbiological outcomes, and patient-centered measures, to improve interpretability of the findings. No critical appraisal or risk-of-bias assessment was performed, as this scoping review aimed to map the available evidence rather than to evaluate the methodological quality of included studies. The included studies were grouped according to intervention categories (mouthrinses, fluoride varnishes, silver diamine fluoride, and glass ionomer–based materials), and the findings were qualitatively synthesized considering outcome types (microbiological, clinical, and patient-centered) and their relevance to settings outside the traditional dental office and community-based settings.

## 3. Results

**Figure 1:**
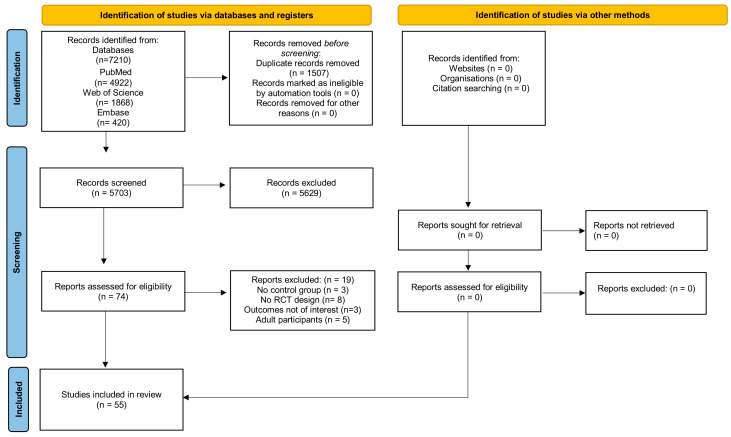
Flow diagram illustrating the study selection process.

In total, 7210 records were retrieved from the electronic databases, comprising PubMed (*n* = 4922), Web of Science (*n* = 1868), and Embase (*n* = 420). The included studies were conducted in a variety of settings, including school-based programs, community initiatives, and clinical environments involving pediatric patients. After deduplication, 5703 references remained and were screened based on the established inclusion criteria. Full-text evaluation was conducted for 74 potentially eligible articles, resulting in the inclusion of 55 studies in the scoping review. Of these, 8 randomized controlled trials (RCTs) investigated mouthrinses, 15 focused on silver diamine fluoride, 16 examined glass ionomer cements, and 16 evaluated fluoride varnishes. The study selection process is illustrated in the flow diagram (Figure 1). Inter-examiner reliability was high, with a kappa coefficient of 0.91.

### 3.1. Mouthrinses

Fluoride mouthrinses demonstrated consistent reductions in the incidence of caries, particularly in supervised, school-based settings. A long-term study showed that weekly 0.2% sodium fluoride mouthrinses reduced DMFS by 34.2% in permanent dentition and 57.5% in primary dentition after 20 months, highlighting a substantial preventive effect in non-fluoridated communities [21]. Similarly, over 3 years of follow-up, discontinuation of supervised fluoride mouthrinse programs resulted in increased caries progression and higher treatment needs, reinforcing the importance of sustained implementation [22].

The duration of effect appears strongly linked to program continuity and supervision, with the most robust outcomes observed in long-term, structured interventions. In contrast, short-term studies (7–30 days) consistently demonstrate significant reductions in *Streptococcus mutans* counts, but without direct evidence of long-term caries reduction [23,24]. For example, fluoride mouthrinse reduced *S. mutans* levels after 30 days, although showing no superiority over alternative formulations such as cranberry-based rinses, suggesting that antimicrobial effects alone may not translate into superior clinical outcomes [23].

Chlorhexidine mouthrinses showed the greatest short-term antimicrobial effect, consistently outperforming alternative formulations in reducing cariogenic bacteria [25,26]. However, these effects are primarily microbiological and short-lived, limiting their clinical relevance for long-term caries prevention. Additionally, studies report similar antimicrobial efficacy between chlorhexidine, fluoride, and probiotic rinses over short- to medium-term follow-up, suggesting that the magnitude of effect may converge over time [23,24].

Combination or alternative formulations, including plant-based or multi-agent rinses, did not demonstrate superior clinical or microbiological outcomes compared to conventional agents, reinforcing that fluoride and chlorhexidine remain the reference standards [25,26].

From a clinical and public health perspective, fluoride mouthrinses stand out as effective, low-cost, and scalable interventions, particularly in school-based programs. Their ability to achieve meaningful reductions in caries incidence over medium-term follow-up supports their use in community settings, especially where access to other fluoride sources is limited [21,27]. However, their effectiveness is highly dependent on supervision, adherence, and long-term program maintenance, which are critical determinants of real-world impact.

Overall, the evidence indicates that fluoride mouthrinses appear to provide sustained clinical benefits when implemented as structured public health interventions, whereas other mouthrinses primarily offer short-term antimicrobial effects with limited evidence of long-term caries reduction.

### 3.2. Fluoridated Varnishes

Fluoride varnishes demonstrated consistent and clinically relevant reductions in caries incidence and progression, with effect sizes varying according to lesion severity, baseline risk, and frequency of application. A community-based study reported that quarterly application of 5% NaF varnish reduced caries incidence by approximately 49% over 24 months, with a higher proportion of children remaining caries-free compared to controls, indicating a substantial preventive effect in high-risk preschool populations [28].

The duration of effect is strongly dependent on repeated applications, with sustained benefits observed in studies with 12–30 months of follow-up. For example, NaF varnish showed moderate caries arrest rates in cavitated dentin lesions and higher effectiveness in moderate lesions after extended follow-up, although performing less effectively than SDF for advanced lesions [28,29]. Similarly, semiannual application resulted in limited but positive effects on dentin caries arrest over 12 months, reinforcing that varnishes are more effective in early-stage lesions than in cavitated dentin lesions [30].

From a clinical relevance perspective, varnishes show their greatest benefit in non-cavitated and early lesions, where they can effectively control lesion progression and promote remineralization. In enamel caries, varnish demonstrated comparable effectiveness to SDF over follow-up periods, with no significant differences in arresting lesion progression [29]. Additionally, varnish showed equivalent prevention of new carious lesions compared to SDF, supporting its role as a non-invasive and esthetically favorable alternative [31].

In contrast, varnishes showed reduced effectiveness in more complex clinical scenarios, such as approximal caries, where no significant difference compared to control conditions was observed after extended follow-up, suggesting that lesion location and baseline severity are key modifiers of treatment effect [32].

Comparative studies indicate that fluoride varnishes provide similar effectiveness to other fluoride delivery systems, supporting flexibility in clinical and public health decision-making. No significant differences were observed between varnish and neutral fluoride gel after 12 months, both reducing caries progression [33,34]. Likewise, varnish showed equivalent effectiveness to glass ionomer sealants in preventing occlusal caries, indicating that intervention choice may depend more on feasibility and patient cooperation than efficacy [13,35].

Regarding formulation, multiple trials consistently demonstrate that conventional 5% NaF varnish performs similarly to modified formulations containing calcium and phosphate, with no additional clinical benefit over follow-up periods, reinforcing its cost-effectiveness and simplicity [36,37].

From a microbiological standpoint, varnishes promote reductions in cariogenic bacteria in short- to medium-term follow-up, often outperforming other chemotherapeutic agents [37,38]. However, as observed in other preventive strategies, these microbiological effects should be interpreted as surrogate outcomes and do not consistently predict long-term clinical outcomes.

In terms of public health applicability, fluoride varnishes offer several advantages: they are safe, well accepted, easy to apply, and do not depend on patient adherence, making them particularly suitable for young children and community-based programs [39,40]. However, their effectiveness may be limited when used as a standalone intervention, as studies show that combining varnish with oral health education results in greater caries prevention than varnish alone, particularly in mixed dentition [32,41].

Overall, fluoride varnishes provide moderate-to-large preventive effects when applied periodically, with the strongest benefits observed in early lesions and high-risk populations. Their impact is maximized when delivered through structured, repeated applications and integrated preventive strategies, supporting their role as a core component of public health caries prevention programs rather than a standalone solution.

### 3.3. Silver Diamine Fluoride (SDF)

SDF has been widely investigated as an effective intervention for controlling cariogenic activity, primarily through the arrest of active caries lesions. Its mechanism of action involves the inhibition of bacterial activity, promotion of remineralization, and modification of the dentin substrate, supporting a minimally invasive approach that is particularly relevant in pediatric populations and in settings with limited access to regular dental care (Table S3). RCTs demonstrate that SDF significantly reduces lesion activity, promotes remineralization of demineralized dentin, and contributes to the stabilization of disease progression in both the short and medium term [42,43,44,45]. Arrest rates of approximately 60–70% have been reported following periodic applications, reinforcing its consistent clinical effectiveness in managing active lesions.

From a microbiological perspective, SDF exhibits potent antibacterial activity, including significant reductions in *S. mutans* and *lactobacilli* in active dentin lesions. However, comparative studies indicate that similar short-term antibacterial effects can be achieved with alternative agents, without significant differences in clinical outcomes [42]. These findings suggest that the clinical effectiveness of SDF cannot be attributed solely to bacterial reduction, but rather to the combined effects of antimicrobial action, remineralization, and structural modification of dentin.

In terms of clinical performance, SDF shows efficacy comparable to, or in some cases greater than, other minimally invasive approaches. When compared with ART, SDF demonstrates similar caries arrest rates, with additional advantages such as reduced clinical time and procedural simplicity [43,46,47]. Combined approaches, such as silver-modified atraumatic restorative techniques, may further improve outcomes over time [46,48].

Importantly, the effects of SDF on caries arrest should be clearly distinguished from its role in caries prevention. While strong and consistent evidence supports its effectiveness in arresting existing lesions; findings related to prevention are more heterogeneous. Clinical trials indicate that SDF demonstrates preventive efficacy comparable to that of fluoride varnishes and sealants, but without consistent evidence of superiority in reducing the incidence of new lesions [31,45]. Therefore, SDF should be primarily interpreted as a caries-arresting agent, and its role in preventing new lesion development should be considered with caution

This distinction is further supported by evidence related to lesion type and stage. SDF shows greater effectiveness in cavitated dentin lesions, whereas in enamel-only lesions it performs similarly to fluoride varnish, without clear clinical advantages [29]. Lesion severity also influences outcomes, with higher arrest rates observed in moderate lesions compared with more advanced stages [49].

Regarding safety and acceptability, available evidence indicates that SDF is safe for use in children, with no association with systemic adverse effects [31,50]. The main limitation is the permanent darkening of arrested lesions, which may affect aesthetic outcomes and influence treatment acceptance, particularly in anterior teeth and in older children. However, caregiver acceptance is generally favorable when clinical benefits are clearly communicated, and the impact on oral health-related quality of life appears limited, with overall improvements driven by pain reduction and disease control [43,47,50,51]. These findings highlight that, although aesthetic darkening is an inherent characteristic of SDF therapy, its influence on treatment acceptance is context-dependent and can be mitigated through appropriate case selection and communication.

### 3.4. Glass Ionomer Cement (GIC)

GIC is established as a consolidated option in contemporary approaches to the prevention and management of dental caries in childhood, mainly within the scope of minimally invasive dentistry and public health. Its indications encompass both preventive actions, such as sealing and control of initial lesions, as well as the treatment of already established carious lesions, including cavitated lesions that require conservative restorative intervention. Its wide use is based on well-established clinical properties, such as chemical adhesion to dental tissue, sustained fluoride release, pulpal biocompatibility, and ease of application in settings with limited infrastructure (Table S4). The literature demonstrates that the use of GIC, alone or associated with atraumatic techniques, constitutes an effective approach for caries control in primary and young permanent [52,53,54]. Within the scope of ART, GIC has been used as the restorative material of choice. Clinical studies have reported high rates of clinical and radiographic success of restorations performed according to ART principles, with maintenance of pulpal vitality and control of lesion progression throughout follow-up [52,53]. In addition, long-term follow-up evaluations present that the effectiveness of ART with GIC is maintained for several years, with lesion inactivation rates exceeding 80% after four years, reinforcing its applicability in primary oral health care programs for children [53,54].

The choice of the type of GIC exerts a significant influence on the clinical performance of ART. In general, clinical findings show that high-viscosity ionomers present better restorative survival rates when compared with lower-cost or lower-viscosity formulations, especially in larger cavities or in posterior primary molars [54,55]. In addition, comparative studies indicate that, among different commercial presentations and manipulation methods, such as encapsulated or manually mixed ionomers, overall restorative survival rates are similar, suggesting that factors related to operative technique and case selection play a determining role in clinical success [49,55].

In proximal cavities and Class II restorations, clinical challenges become more evident. Restorative survival rates are lower when compared with simple occlusal restorations, regardless of the type of ionomer used [49,55,56]. Specific technical strategies, such as the creation of proximal retentions, have been evaluated as a means to increase restorative longevity, with the scientific literature reporting a significant improvement in success rates in short- and medium-term follow-ups [56,57].

In addition to its restorative use, GIC also presents applicability as a sealing material in preventive strategies. RCTs shown that ionomer-based sealants applied according to ART principles are effective in preventing dentinal caries lesions in newly erupted permanent molars, exhibiting a preventive effect similar to that of other fluoride-based approaches, even when material retention is partial or temporary [58,59]. These results help to corroborate that the preventive effect of GIC does not depend exclusively on physical retention, but also on continuous fluoride release and modulation of the cariogenic environment. In populations with specific clinical conditions, such as molars affected by molar–incisor hypomineralization, the use of GIC sealant techniques exerts a protective effect against the development of dental caries, although it does not show similar effectiveness in preventing post-eruptive fracture [58,60]. These findings contribute to clinical guidelines by noting that this material has specific limits of intervention, particularly in tissues with severe structural compromise. As well as, the pulpal safety of restorations with GIC has also been documented. RCTs in primary teeth with deep lesions demonstrate that restorations performed exclusively with high-viscosity ionomer present pulpal vitality rates similar to those obtained with techniques that include calcium hydroxide lining, in addition to higher restorative survival rates over the follow-up period [52,61]. These findings reinforce the suitability of GIC as a definitive restorative material in minimally invasive approaches.

In recent years, the association of GIC with silver diamine fluoride, through the technique known as silver-modified atraumatic restorative treatment (SMART), has been the subject of increasing investigation. RCTs support those SMART shows clinical performance similar to conventional ART in terms of restorative survival, both in the short and medium term [62,63]. In addition, comparative studies point to relevant operational advantages, including shorter clinical time, greater acceptability among children, and a significant reduction in total costs per restoration, aspects of great relevance in the context of public health [62,63,64].

From a behavioral standpoint, the use of atraumatic approaches with GIC has been associated with beneficial outcomes in managing childhood anxiety. Clinical trials reveal better modulation of physiological stress markers during dental care, markedly when ART is combined with oral health educational strategies [64,65].

## 4. Discussion

Despite substantial advances in the development of preventive materials and techniques aimed at controlling dental caries in childhood, relevant limitations persist when these interventions are analyzed in the context of minimally invasive dentistry applied outside the traditional outpatient setting. The included studies represent a heterogeneous mix of settings, including school-based programs, community initiatives, and clinical environments involving pediatric patients. These sources of heterogeneity as differences in clinical setting, dentition stage, lesion severity, intervention protocols, and follow-up duration; limit direct comparability between studies and should be considered when interpreting the findings. Although this review emphasizes public health and community-based perspectives, not all included studies were conducted exclusively in non-clinical settings. Therefore, the applicability of the findings to public health contexts should be interpreted with caution, as the effectiveness and feasibility of these interventions may vary depending on the implementation setting. Differences in implementation settings, including clinical, school-based, and community environments, may influence both the effectiveness and feasibility of interventions, thereby limiting the direct transferability of findings across contexts. In school programs, daycare centers, and community actions, the effectiveness of strategies such as fluoridated mouthrinses, varnishes, SDF, and GICs is strongly conditioned not only by the children’s baseline caries risk, but also by the operational conditions of the setting, the socioeconomic context, and the possibility of longitudinal follow-up. It is important to distinguish between caries’ arrest and caries’ prevention, as these represent different therapeutic objectives. While some minimally invasive interventions are effective in arresting existing lesions, their effectiveness in preventing the development of new lesions is more variable and context dependent. In these situations, the punctual or isolated application of these interventions tends to produce limited effects, specifically in populations with a high cariogenic challenge, requiring greater frequency of reapplication and integration with educational and behavioral actions for clinical benefits to be sustained over time.

One of the main challenges of preventive practice in field settings relates to adherence and protocol standardization. Unlike controlled clinical environments, school- and community-based programs are affected by absenteeism, student turnover, interruptions in the school calendar, infrastructure limitations, and constraints in human resources. These factors compromise the regularity of interventions and hinder the maintenance of preventive protocols based on periodic reapplications, ultimately reducing the predictability of clinical outcomes.

In addition, a substantial proportion of the available evidence still focuses on intermediate outcomes, such as microbial reduction, lesion arrest, or control of white spot lesions, while fewer studies provide long-term follow-up capable of demonstrating sustained effects on caries experience at the population level. Importantly, the different types of outcomes reported in the included studies—clinical, microbiological (surrogate), operational, and patient-centered—should not be interpreted as equivalent, as they reflect distinct dimensions of intervention performance and carry different levels of clinical relevance. Furthermore, the absence of a formal risk-of-bias assessment limits the ability to determine the internal validity of the included studies and may have influenced the interpretation of reported clinical effects.

Although several systematic reviews have evaluated the clinical efficacy of minimally invasive interventions such as silver diamine fluoride, fluoride varnishes, and atraumatic restorative treatment, these studies are predominantly based on controlled or clinical settings. In contrast, the present review contributes to highlighting how the effectiveness of these interventions is strongly influenced by real-world implementation conditions, particularly in community-based and public health contexts. By focusing on operational variability, adherence challenges, and contextual constraints, this work provides a complementary perspective that goes beyond efficacy estimates and supports decision-making in non-clinical environments.

Clinical and social acceptance also play a critical role in the success of minimally invasive approaches outside traditional clinical settings. Although silver diamine fluoride is highly effective in arresting cavitated lesions, the permanent dark staining of treated areas may negatively influence acceptance, especially in anterior teeth. Similarly, chlorhexidine-based mouthrinses, despite their antimicrobial efficacy, are associated with adverse effects such as dental staining and taste alteration, limiting their long-term applicability in pediatric populations.

The effectiveness of fluoridated varnishes and mouthrinses is also context-dependent, particularly in newly erupted permanent teeth, where outcomes tend to be more favorable when these interventions are combined with complementary strategies, such as occlusal sealants and structured oral health education. In younger children, the use of fluoridated products requires careful indication and supervision due to the risk of ingestion, fluorosis, and limited ability to expect.

Glass ionomer cements present clear operational advantages for field use, including ease of application, chemical adhesion, and relative tolerance to moisture. However, their longevity may be reduced in extensive cavities or areas subjected to high functional load, reinforcing the importance of appropriate case selection and caries risk stratification.

Taken together, these findings support the notion that minimally invasive interventions should not be considered isolated solutions, but rather as components of integrated, risk-based strategies. The literature increasingly suggests that combining preventive measures with educational and behavioral approaches leads to more consistent and sustainable outcomes, particularly in high-risk and socially vulnerable populations.

As the understanding of dental caries as a biofilm-dependent condition, modulated by behavioral, social, and environmental factors, expands, preventive strategies in childhood tend to move away from isolated interventions and incorporate more integrated and contextually appropriate models. The control of dental caries in children becomes grounded in the rational combination of different preventive and minimally invasive approaches, articulating topical methods, continuous educational actions, and strategies adapted to individual and collective risk. The available literature shows that the association between fluoride varnishes, SDF, restorative materials, and continuous oral health education programs tends to produce more consistent results than the isolated application of any single method. In this sense, the adoption of risk-stratified preventive models emerges as a promising alternative, allowing resources and interventions to be directed according to lesion stage, type of dentition, and the social vulnerability of children. Accordingly, practice outside the dental office ceases to be seen as a secondary approach and comes to play an important role in the organization of public policies aimed at childhood.

The development of new materials also represents a field of growing interest for preventive practice in field settings. Bioactive formulations capable of releasing fluoride in a controlled manner, incorporating selective antimicrobial agents, and promoting remineralization processes. These innovations have the potential to reduce the need for frequent reapplications and to enhance clinical effectiveness in contexts with difficult longitudinal follow-up, such as school programs. Thus, initial lesions can be managed primarily with varnishes and educational actions, while cavitated lesions can be controlled through SDF or atraumatic restorative approaches, respecting the operational limits of the extra-clinical environment.

In addition, the need to expand the conduct of studies that assess not only clinical efficacy, but also cost-effectiveness, sustainability, and the population impact of these interventions is becoming increasingly evident. The consolidation of caries prevention as a public policy depends on the integration between oral health teams, managers, and the school community, as well as on the strengthening of actions directed at critical periods of dental development, such as early childhood and the eruption of permanent molars. Thus, the expansion of the preventive scope and multiprofessional training become key components to ensure more equitable, resolutive interventions aligned with the principles of minimally invasive dentistry.

## 5. Conclusions

The findings of this scoping review suggest that minimally invasive dentistry (MID) approaches have been investigated as potential strategies for the prevention and management of dental caries in childhood within public health and community-based settings. Reported clinical outcomes vary according to lesion stage, intervention context, and follow-up conditions, indicating that isolated measures may not be sufficient. While integrated, risk-oriented models have been proposed, the strength of these conclusions is limited. The heterogeneity of interventions and outcome measures, the predominance of short-term follow-up and surrogate microbiological outcomes, and the absence of formal quality appraisal reduce the reliability and applicability of the evidence. Consequently, this synthesis provides a descriptive overview rather than definitive conclusions on effectiveness or public health relevance. Further research with rigorous methodological quality assessment, standardized clinical outcomes, and longer follow-up is required to substantiate these preliminary observations.

## Data Availability

No new data was created or analyzed in this study. Data sharing is not applicable to this article.

## Supplementary Materials

The following supporting information can be downloaded at: https://www.mdpi.com/article/10.3390/healthcare14091155/s1, Table S1: Information sources and search strategy; Table S2: General characteristics of the included studies evaluating the effects of mouthwashes on dental caries management; Table S3: General characteristics of the included studies evaluating the effects of fluoride varnish on dental caries management; Table S4: General characteristics of the included studies evaluating the effects of silver diamine fluoride on dental caries management; Table S5: General characteristics of the included studies evaluating the effects of glass ionomer cement on dental caries management.

## Abbreviations

The following abbreviations are used in this manuscript:

ART	Atraumatic Restorative Treatment
ECC	Early Childhood Caries
GIC	Glass Ionomer Cements
MID	Minimally Invasive Dentistry
PCC	Population, Concept, and Context
PRISMA-ScR	Preferred Reporting Items for Systematic Reviews and Meta-Analyses extension for Scoping Reviews
RCTs	Randomized Clinical Trials
SDF	Silver Diamine Fluoride
SMART	Silver-modified Atraumatic Restorative Treatment
