# Supplementary material for: Minimally Invasive Interventions for Childhood Caries: A Scoping Review of Their Applicability in Public Health and Community Settings
Source: Healthcare (Basel). 2026 Apr 25;14(9):1155. doi: 10.3390/healthcare14091155 (PMC13163313; doi:10.3390/healthcare14091155)
Supplement: Supplementary file 1 [file healthcare-14-01155-s001.zip › healthcare-4175742-supplementary.pdf]

Literature Review Methodology

Table 1. Information sources and search strategy

This review was conducted in accordance with the Preferred Reporting Items for Systematic Reviews and Meta-Analyses extension for Scoping Reviews (PRISMA-ScR) guidelines [18] and was informed by methodological approaches described in recent publications [19,20].

**Search Strategy**
**MOUTHRINSE: 1294**
**PUBMED: 1050**<br>(((Mouth Wash) OR (Wash, Mouth) OR (Mouth Rinse) OR (Mouth Rinses) OR (Rinse, Mouth) OR (Rinses, Mouth) OR (Mouth Bath) OR (Bath, Mouth) OR (Baths, Mouth) OR (Mouth Baths) OR (Chlorhexidine mouthrinse) OR (Fluoride mouthrinse) AND ((Child) OR (Preschool Child) OR (Children) OR (Preschool) OR (Preschool Children) OR (early childhood caries) OR (Pediatric Dentistry) OR (Infant) OR (Child care) OR (Infant care))) AND ((Initial caries) OR (incipient caries) OR (early caries) OR (Dental Decay) OR (Dental Cavities) OR (Cavities, Dental) OR (Cavity, Dental) OR (Decay, Dental) OR (Carious Lesions) OR (Carious Lesion) OR (Lesion, Carious) OR (Lesions, Carious) OR (Caries, Dental) OR (Dental Cavity) OR (Dental White Spot) OR (Spot, Dental White) OR (Spots, Dental White) OR (Dental White Spots) OR (White Spot, Dental) OR (White Spots, Dental) OR (Carious Dentin) OR (Carious Dentins) OR (Dentin, Carious) OR (Dentins, Carious))<br><br>(http://www.ncbi.nlm.nih.gov/pubmed)
**WEB OF SCIENCE: 199**<br>(Mouth Wash) OR (Wash, Mouth) OR (Mouth Rinse) OR (Mouth Rinses) OR (Rinse, Mouth) OR (Rinses, Mouth) OR (Mouth Bath) OR (Bath, Mouth) OR (Baths, Mouth) OR (Mouth Baths) OR (Chlorhexidine Mouthrinse) OR (Fluoride Mouthrinse) (Topic) and (Child) OR (Preschool Child) OR (Children) OR (Preschool) OR (Preschool Children) OR (early childhood caries) OR (Pediatric Dentistry) OR (Infant) OR (Child care) OR (Infant care) (Topic) and (Initial caries) OR (incipient caries) OR (early caries) OR (Dental Decay) OR (Dental Cavities) OR (Cavities, Dental) OR (Cavity, Dental) OR (Decay, Dental) OR (Carious Lesions) OR (Carious Lesion) OR (Lesion, Carious) OR (Lesions, Carious) OR (Caries, Dental) OR (Dental Cavity) OR (Dental White Spot) OR (Spot, Dental White) OR (Spots, Dental White) OR (Dental White Spots) OR (White Spot, Dental) OR (White Spots, Dental) OR (Carious Dentin) OR (Carious dentine) OR (Dentin, Carious) OR (dentine, Carious)<br><br>(https://clarivate.com/webofsciencegroup/solutions/web-of-science-core-collection)
**EMBASE: 45**<br>('mouth wash'/exp OR 'mouth wash' OR (('mouth'/exp OR mouth) AND wash) OR 'wash, mouth' OR (wash, AND ('mouth'/exp OR mouth)) OR 'mouth rinse'/exp OR 'mouth rinse' OR (('mouth'/exp OR mouth) AND rinse) OR 'mouth rinses'/exp OR 'mouth rinses' OR (('mouth'/exp OR mouth) AND rinses) OR 'rinse, mouth' OR (rinse, AND ('mouth'/exp OR mouth)) OR 'rinses, mouth' OR (rinses, AND ('mouth'/exp OR mouth)) OR 'mouth bath' OR (('mouth'/exp OR mouth) AND ('bath'/exp OR bath)) OR 'bath, mouth' OR (('bath,'/exp OR bath,) AND ('mouth'/exp OR mouth)) OR 'baths, mouth' OR (('baths,'/exp OR baths,) AND ('mouth'/exp OR mouth)) OR 'mouth baths' OR (('mouth'/exp OR mouth) AND ('baths'/exp OR baths)) OR 'chlorhexidine mouthrinse' OR (('chlorhexidine'/exp OR chlorhexidine) AND ('mouthrinse'/exp OR mouthrinse)) OR 'fluoride mouthrinse' OR (('fluoride'/exp OR fluoride) AND ('mouthrinse'/exp OR mouthrinse))) AND (child:ti,ab,kw OR 'preschool child':ti,ab,kw OR children:ti,ab,kw OR preschool:ti,ab,kw OR 'preschool children':ti,ab,kw OR 'early childhood caries':ti,ab,kw OR 'pediatric dentistry':ti,ab,kw OR infant:ti,ab,kw OR 'child care':ti,ab,kw OR 'infant care':ti,ab,kw) AND ('initial caries':ti,ab,kw OR 'incipient caries':ti,ab,kw OR 'early caries':ti,ab,kw OR 'dental decay':ti,ab,kw OR 'dental cavities':ti,ab,kw OR 'cavities, dental':ti,ab,kw OR 'cavity, dental':ti,ab,kw OR 'decay, dental':ti,ab,kw OR

'carious lesions':ti,ab,kw OR 'carious lesion':ti,ab,kw OR 'lesion, carious':ti,ab,kw OR 'lesions, carious':ti,ab,kw OR 'caries, dental':ti,ab,kw OR 'dental cavity':ti,ab,kw OR 'dental white spot':ti,ab,kw OR 'spot, dental white':ti,ab,kw OR 'spots, dental white':ti,ab,kw OR 'dental white spots':ti,ab,kw OR 'white spot, dental':ti,ab,kw OR 'white spots, dental':ti,ab,kw OR 'carious dentin':ti,ab,kw OR 'carious dentins':ti,ab,kw OR 'dentin, carious':ti,ab,kw OR 'dentins, carious':ti,ab,kw)

(https://www.embase.com)

FLUORIDE VARNISH: 3054

PUBMED: 2141

((Fluoride Varnishes) OR (Varnishes, Fluoride) OR (calcium fluoride - sodium fluoride varnish) OR (CaF2-NaF varnish)) AND ((Child) OR (Preschool Child) OR (Children) OR (Preschool) OR (Preschool Children) OR (early childhood caries) OR (Pediatric Dentistry) OR (deciduous teeth) OR (Infant) OR (Child care) OR (Infant care))) AND ((initial caries) OR (incipient caries) OR (early caries) OR (Dental Decay) OR (Dental Cavities) OR (Cavities, Dental) OR (Cavity, Dental) OR (Decay, Dental) OR (Carious Lesions) OR (Carious Lesion) OR (Lesion, Carious) OR (Lesions, Carious) OR (Caries, Dental) OR (Dental Cavity) OR (Dental White Spot) OR (Spot, Dental White) OR (Spots, Dental White) OR (Dental White Spots) OR (White Spot, Dental) OR (White Spots, Dental) OR (Carious Dentin) OR (Carious Dentins) OR (Dentin, Carious) OR (Dentins, Carious))

(http://www.ncbi.nlm.nih.gov/pubmed)

WEB OF SCIENCE: 870

(Fluoride Varnishes) OR (Varnishes, Fluoride) OR (calcium fluoride - sodium fluoride varnish) OR (CaF2-NaF varnish) (Topic) and (Child) OR (Preschool Child) OR (Children) OR (Preschool) OR (Preschool Children) OR (early childhood caries) OR (Pediatric Dentistry) OR (Infant) OR (Child care) OR (Infant care) (Topic) and (Initial caries) OR (incipient caries) OR (early caries) OR (Dental Decay) OR (Dental Cavities) OR (Cavities, Dental) OR (Cavity, Dental) OR (Decay, Dental) OR (Carious Lesions) OR (Carious Lesion) OR (Lesion, Carious) OR (Lesions, Carious) OR (Caries, Dental) OR (Dental Cavity) OR (Dental White Spot) OR (Spot, Dental White) OR (Spots, Dental White) OR (Dental White Spots) OR (White Spot, Dental) OR (White Spots, Dental) OR (Carious Dentin) OR (Carious dentine) OR (Dentin, Carious) OR (dentine, Carious)

(https://clarivate.com/webofsciencegroup/solutions/web-of-science-core-collection)

EMBASE: 43

('fluoride varnishes' OR (('fluoride'/exp OR fluoride) AND varnishes) OR 'varnishes, fluoride' OR (varnishes, AND ('fluoride'/exp OR fluoride)) OR 'calcium fluoride - sodium fluoride varnish' OR (('calcium'/exp OR calcium) AND - AND ('sodium'/exp OR sodium) AND ('fluoride'/exp OR fluoride) AND ('varnish'/exp OR varnish)) OR 'caf2-naf varnish' OR ('caf2 naf' AND ('varnish'/exp OR varnish))) AND (child:ti,ab,kw OR 'preschool child':ti,ab,kw OR children:ti,ab,kw OR preschool:ti,ab,kw OR 'preschool children':ti,ab,kw OR 'early childhood caries':ti,ab,kw OR 'pediatric dentistry':ti,ab,kw OR infant:ti,ab,kw OR 'child care':ti,ab,kw OR 'infant care':ti,ab,kw) AND ('initial caries':ti,ab,kw OR 'incipient caries':ti,ab,kw OR 'early caries':ti,ab,kw OR 'dental decay':ti,ab,kw OR 'dental cavities':ti,ab,kw OR 'cavities, dental':ti,ab,kw OR 'cavity, dental':ti,ab,kw OR 'decay, dental':ti,ab,kw OR 'carious lesions':ti,ab,kw OR 'carious lesion':ti,ab,kw OR 'lesion, carious':ti,ab,kw OR 'lesions, carious':ti,ab,kw OR 'caries, dental':ti,ab,kw OR 'dental cavity':ti,ab,kw OR 'dental white spot':ti,ab,kw OR 'spot, dental white':ti,ab,kw OR 'spots, dental white':ti,ab,kw OR 'dental white spots':ti,ab,kw OR 'white spot, dental':ti,ab,kw OR 'white spots, dental':ti,ab,kw OR 'carious dentin':ti,ab,kw OR 'carious dentins':ti,ab,kw OR 'dentin, carious':ti,ab,kw OR 'dentins, carious':ti,ab,kw)

(https://www.embase.com)

SILVER DIAMINE FLUORIDE: 1212

PUBMED: 601

(((Silver diamine fluoride) OR (SDF) OR (Nano silver fluoride) OR (silver ammonia fluoride) OR (Silver nitrate) OR (Silver-modified atraumatic restorative technique)) AND ((Child) OR (Preschool Child) OR (Children) OR (Preschool) OR (Preschool Children) OR (early childhood caries) OR (Pediatric Dentistry) OR (deciduous teeth) OR (Infant) OR (Child care) OR (Infant care))) AND ((initial caries) OR (incipient caries) OR (early caries) OR (Dental Decay) OR (Dental Cavities) OR (Cavities, Dental) OR (Cavity, Dental) OR (Decay, Dental) OR (Carious Lesions) OR (Carious Lesion) OR (Lesion, Carious) OR (Lesions, Carious) OR (Caries, Dental) OR (Dental Cavity) OR (Dental White Spot) OR (Spot, Dental White) OR (Spots, Dental White) OR (Dental White Spots) OR (White Spot, Dental) OR (White Spots, Dental) OR (Carious Dentin) OR (Carious Dentins) OR (Dentin, Carious) OR (Dentins, Carious))

(http://www.ncbi.nlm.nih.gov/pubmed)

WEB OF SCIENCE: 458

(Silver diamine fluoride) OR (SDF) OR (Nano silver fluoride) OR (silver ammonia fluoride) OR (Silver nitrate) OR (Silver-modified atraumatic restorative technique) (Topic) and (Child) OR (Preschool Child) OR (Children) OR (Preschool) OR (Preschool Children) OR (early childhood caries) OR (Pediatric Dentistry) OR (Infant) OR (Child care) OR (Infant care) (Topic) and (Initial caries) OR (incipient caries) OR (early caries) OR (Dental Decay) OR (Dental Cavities) OR (Cavities, Dental) OR (Cavity, Dental) OR (Decay, Dental) OR (Carious Lesions) OR (Carious Lesion) OR (Lesion, Carious) OR (Lesions, Carious) OR (Caries, Dental) OR (Dental Cavity) OR (Dental White Spot) OR (Spot, Dental White) OR (Spots, Dental White) OR (Dental White Spots) OR (White Spot, Dental) OR (White Spots, Dental) OR (Carious Dentin) OR (Carious dentine) OR (Dentin, Carious) OR (dentine, Carious)

(https://clarivate.com/webofsciencegroup/solutions/web-of-science-core-collection)

EMBASE: 153

('silver diamine fluoride'/exp OR 'silver diamine fluoride' OR (('silver'/exp OR silver) AND ('diamine'/exp OR diamine) AND ('fluoride'/exp OR fluoride)) OR sdf OR 'nano silver fluoride' OR (('nano'/exp OR nano) AND ('silver'/exp OR silver) AND ('fluoride'/exp OR fluoride)) OR 'silver ammonia fluoride' OR (('silver'/exp OR silver) AND ('ammonia'/exp OR ammonia) AND ('fluoride'/exp OR fluoride)) OR 'silver nitrate'/exp OR 'silver nitrate' OR (('silver'/exp OR silver) AND ('nitrate'/exp OR nitrate)) OR 'silver-modified atraumatic restorative technique' OR ('silver modified' AND atraumatic AND restorative AND ('technique'/exp OR technique))) AND (child:ti,ab,kw OR 'preschool child':ti,ab,kw OR children:ti,ab,kw OR preschool:ti,ab,kw OR 'preschool children':ti,ab,kw OR 'early childhood caries':ti,ab,kw OR 'pediatric dentistry':ti,ab,kw OR infant:ti,ab,kw OR 'child care':ti,ab,kw OR 'infant care':ti,ab,kw) AND ('initial caries':ti,ab,kw OR 'incipient caries':ti,ab,kw OR 'early caries':ti,ab,kw OR 'dental decay':ti,ab,kw OR 'dental cavities':ti,ab,kw OR 'cavities, dental':ti,ab,kw OR 'cavity, dental':ti,ab,kw OR 'decay, dental':ti,ab,kw OR 'carious lesions':ti,ab,kw OR 'carious lesion':ti,ab,kw OR 'lesion, carious':ti,ab,kw OR 'lesions, carious':ti,ab,kw OR 'caries, dental':ti,ab,kw OR 'dental cavity':ti,ab,kw OR 'dental white spot':ti,ab,kw OR 'spot, dental white':ti,ab,kw OR 'spots, dental white':ti,ab,kw OR 'dental white spots':ti,ab,kw OR 'white spot, dental':ti,ab,kw OR 'white spots, dental':ti,ab,kw OR 'carious dentin':ti,ab,kw OR 'carious dentins':ti,ab,kw OR 'dentin, carious':ti,ab,kw OR 'dentins, carious':ti,ab,kw)

(https://www.embase.com)

GLASS IONOMER CEMENT: 1650

PUBMED: 1130

(((Glass Ionomer Cement) OR (Cement, Glass Ionomer) OR (Cements, Glass Ionomer) OR (Ionomer Cement, Glass) OR (Ionomer cement, glass) OR (resin modified glass ionomer cement) OR (resin modified glass ionomer cements) OR (resin modified glass ionomer luting) OR (resin modified glass ionomer luting cement) OR (resin reinforced glass ionomer) OR (resin reinforced glass ionomer cement) OR (Polyalkenoate Cement) OR (Cement, Polyalkenoate) OR (Cements, Polyalkenoate) OR (Glass Polyalkenoate Cement) OR (Cement, Glass Polyalkenoate) OR (Cements, Glass Polyalkenoate) OR (Polyalkenoate Cement, Glass) OR (Glass-Ionomer Cement) OR (Cement, Glass-Ionomer) OR (Cements, Glass-Ionomer) OR (Glass-Ionomer Cements) OR (Glass Polyalkenoate Cements) OR (Polyalkenoate Cements)) AND ((Child) OR (Preschool Child) OR (Children) OR (Preschool) OR (Preschool Children) OR (early childhood caries) OR (Pediatric Dentistry) OR (Infant) OR (Child care) OR (Infant care))) AND ((Initial caries) OR

(incipient caries) OR (early caries) OR (Dental Decay) OR (Dental Cavities) OR (Cavities, Dental) OR (Cavity, Dental) OR (Decay, Dental) OR (Carious Lesions) OR (Carious Lesion) OR (Lesion, Carious) OR (Lesions, Carious) OR (Caries, Dental) OR (Dental Cavity) OR (Dental White Spot) OR (Spot, Dental White) OR (Spots, Dental White) OR (Dental White Spots) OR (White Spot, Dental) OR (White Spots, Dental) OR (Carious Dentin) OR (Carious Dentins) OR (Dentin, Carious) OR (Dentins, Carious))

(http://www.ncbi.nlm.nih.gov/pubmed)

WEB OF SCIENCE: 341

(Glass Ionomer Cement) OR (Cement, Glass Ionomer) OR (Cements, Glass Ionomer) OR (Ionomer Cement, Glass) OR (Ionomer cement, glass) OR (resin modified glass ionomer cement) OR (resin modified glass ionomer cements) OR (resin modified glass ionomer luting) OR (resin modified glass ionomer luting cement) OR (resin reinforced glass ionomer) OR (resin reinforced glass ionomer cement) OR (Polyalkenoate Cement) OR (Cement, Polyalkenoate) OR (Cements, Polyalkenoate) OR (Glass Polyalkenoate Cement) OR (Cement, Glass Polyalkenoate) OR (Cements, Glass Polyalkenoate) OR (Polyalkenoate Cement, Glass) OR (Glass-Ionomer Cement) OR (Cement, Glass-Ionomer) OR (Cements, Glass-Ionomer) OR (Glass-Ionomer Cements) OR (Glass Polyalkenoate Cements) OR (Polyalkenoate Cements) (Topic) and (Child) OR (Preschool Child) OR (Children) OR (Preschool) OR (Preschool Children) OR (early childhood caries) OR (Pediatric Dentistry) OR (Infant) OR (Child care) OR (Infant care) (Topic) and (Initial caries) OR (incipient caries) OR (early caries) OR (Dental Decay) OR (Dental Cavities) OR (Cavities, Dental) OR (Cavity, Dental) OR (Decay, Dental) OR (Carious Lesions) OR (Carious Lesion) OR (Lesion, Carious) OR (Lesions, Carious) OR (Caries, Dental) OR (Dental Cavity) OR (Dental White Spot) OR (Spot, Dental White) OR (Spots, Dental White) OR (Dental White Spots) OR (White Spot, Dental) OR (White Spots, Dental) OR (Carious Dentin) OR (Carious dentine) OR (Dentin, Carious) OR (dentine, Carious)

(https://clarivate.com/webofsciencegroup/solutions/web-of-science-core-collection)

EMBASE: 179

('glass ionomer cement'/exp OR 'glass ionomer cement' OR (('glass'/exp OR glass) AND ('ionomer'/exp OR ionomer) AND ('cement'/exp OR cement)) OR 'cement, glass ionomer'/exp OR 'cement, glass ionomer' OR (('cement,'/exp OR cement,) AND ('glass'/exp OR glass) AND ('ionomer'/exp OR ionomer)) OR 'cements, glass ionomer' OR (cements, AND ('glass'/exp OR glass) AND ('ionomer'/exp OR ionomer)) OR 'ionomer cement, glass' OR (('ionomer'/exp OR ionomer) AND ('cement,'/exp OR cement,) AND ('glass'/exp OR glass)) OR 'resin modified glass ionomer cement'/exp OR 'resin modified glass ionomer cement' OR (('resin'/exp OR resin) AND modified AND ('glass'/exp OR glass) AND ('ionomer'/exp OR ionomer) AND ('cement'/exp OR cement)) OR 'resin modified glass ionomer cements' OR (('resin'/exp OR resin) AND modified AND ('glass'/exp OR glass) AND ('ionomer'/exp OR ionomer) AND cements) OR 'resin modified glass ionomer luting' OR (('resin'/exp OR resin) AND modified AND ('glass'/exp OR glass) AND ('ionomer'/exp OR ionomer) AND luting) OR 'resin modified glass ionomer luting cement' OR (('resin'/exp OR resin) AND modified AND ('glass'/exp OR glass) AND ('ionomer'/exp OR ionomer) AND luting AND ('cement'/exp OR cement)) OR 'resin reinforced glass ionomer' OR (('resin'/exp OR resin) AND reinforced AND ('glass'/exp OR glass) AND ('ionomer'/exp OR ionomer)) OR 'resin reinforced glass ionomer cement' OR (('resin'/exp OR resin) AND reinforced AND ('glass'/exp OR glass) AND ('ionomer'/exp OR ionomer) AND ('cement'/exp OR cement)) OR 'polyalkenoate cement' OR (polyalkenoate AND ('cement'/exp OR cement)) OR 'cement, polyalkenoate' OR (('cement,'/exp OR cement,) AND polyalkenoate) OR 'cements, polyalkenoate' OR (cements, AND polyalkenoate) OR 'glass polyalkenoate cement'/exp OR 'glass polyalkenoate cement' OR (('glass'/exp OR glass) AND polyalkenoate AND ('cement'/exp OR cement)) OR 'cement, glass polyalkenoate' OR (('cement,'/exp OR cement,) AND ('glass'/exp OR glass) AND polyalkenoate) OR 'cements, glass polyalkenoate' OR (cements, AND ('glass'/exp OR glass) AND polyalkenoate) OR 'polyalkenoate cement, glass' OR (polyalkenoate AND ('cement,'/exp OR cement,) AND ('glass'/exp OR glass)) OR 'glass-ionomer cement'/exp OR 'glass-ionomer cement' OR (('glass ionomer'/exp OR 'glass ionomer') AND ('cement'/exp OR cement)) OR 'cement, glass-ionomer'/exp OR 'cement, glass-ionomer' OR (('cement,'/exp OR cement,) AND ('glass ionomer'/exp OR 'glass ionomer')) OR 'cements, glass-ionomer' OR (cements, AND ('glass ionomer'/exp OR 'glass ionomer')) OR 'glass-ionomer cements'/exp OR 'glass-ionomer cements' OR (('glass ionomer'/exp OR 'glass ionomer') AND cements) OR 'glass polyalkenoate cements' OR (('glass'/exp OR glass) AND polyalkenoate AND cements) OR 'polyalkenoate cements' OR (polyalkenoate AND cements)) AND (child:ti,ab,kw OR 'preschool child':ti,ab,kw OR children:ti,ab,kw OR preschool:ti,ab,kw OR 'preschool children':ti,ab,kw OR 'early childhood caries':ti,ab,kw OR 'pediatric dentistry':ti,ab,kw OR infant:ti,ab,kw OR 'child care':ti,ab,kw OR 'infant care':ti,ab,kw) AND ('initial caries':ti,ab,kw OR

'incipient caries':ti,ab,kw OR 'early caries':ti,ab,kw OR 'dental decay':ti,ab,kw OR 'dental cavities':ti,ab,kw OR 'cavities, dental':ti,ab,kw OR 'cavity, dental':ti,ab,kw OR 'decay, dental':ti,ab,kw OR 'carious lesions':ti,ab,kw OR 'carious lesion':ti,ab,kw OR 'lesion, carious':ti,ab,kw OR 'lesions, carious':ti,ab,kw OR 'caries, dental':ti,ab,kw OR 'dental cavity':ti,ab,kw OR 'dental white spot':ti,ab,kw OR 'spot, dental white':ti,ab,kw OR 'spots, dental white':ti,ab,kw OR 'dental white spots':ti,ab,kw OR 'white spot, dental':ti,ab,kw OR 'white spots, dental':ti,ab,kw OR 'carious dentin':ti,ab,kw OR 'carious dentins':ti,ab,kw OR 'dentin, carious':ti,ab,kw OR 'dentins, carious':ti,ab,kw)

(https://www.embase.com)

Table 2. General characteristics of the included studies evaluating the effects of mouthwashes on dental caries management.

Author/ year (Country)	Population (N, Age, Gen-der)	Interventions	Protocol	Assessment Method	Follow-up	Main results
Bansal et al., 2024 (India)	280 8-12 years	G1: Cranberry mouth rinse containing 3 mg/ml non-dialyzable material<br><br>G2: 0.05% sodium fluoride mouth rinse.	G1: 10 mL cranberry rinse (3 mg/mL), once daily, 1 min, 30 days (supervised)<br>G2: 10 mL 0.05% NaF rinse, same protocol	Dental plaque collected before and after 30 days: CFU/ml	1 month	Both groups reduced *S. mutans*; no significant difference
Gedam et al., 2022 (India)	51 8- 12 years	G1: Mouthrinses containing 0.12% chlorhexidine (CHX)<br><br>G2: Mouthrinses containing 0.05% sodium fluoride (NaF)<br>G3: Probiotic mouth rinse (PB)	G1: 10 mL rinse, 1 min, twice daily, 2-week phases (under parental supervision): CHX (0.12%);<br>G2: 10 mL rinse, 1 min, twice daily, 2-week phases (under parental supervision): NaF (0.05%);<br>G3: 10 mL rinse, 1 min, twice daily, 2-week phases (under parental supervision): Probiotic.	CFU/mL, Taste acceptance and adherence to use.	3 and 6 months	Similar antimicrobial effect; CHX showed faster reduction
Patila et al., 2019 (India)	30 8-13 years	G1: Probiotic milk (Yakult) containing 6.5 billion Lactobacillus casei<br>G2: Fluoride mouthwash (NaF 0.044% = 200 ppm F^-)	G1: Probiotic milk (10 mL), once daily, 7 days<br>G2: NaF rinse (0.044%), same protocol	CFU/mL + Plaque Index	7 days	After 7 days, both groups reduced *S. mutans* and plaque index, with no significant difference; fluoride mouthwash showed comparable antimicrobial

						efficacy to probiotic milk.
Hegde et al., 2017 (India)	75<br>8-12 years	G1: 0.12% Chlorhexidine<br>G2: Mouthrinse combined with 0.05% NaF and 0.2% CHX; G3: 0.5% green tea extract	All children used 10 mL of mouthrinse for 1 min once daily for 2 weeks after breakfast, under supervision (school and parents); saliva samples were collected at baseline and after 2 weeks.	CFUs in saliva were measured before and after the intervention.	14 days	0.12% CHX mouthrinse showed the greatest reduction in *S. mutans* and Lactobacillus compared to combination and green tea rinses, confirming its strong short-term antimicrobial efficacy.
Lakade et al., 2014 (India)	30<br>8-10 years	G1: Mouthrinse 0.2% chlorhexidine gluconate<br>G2: Mouthrise 0.03% triclosan, 0.05% sodium fluoride, and 5% xylitol	G1: 10 mL of 0.2% CHX, 1 min, twice daily for 15 days (under parental supervision); no food/drink for 30 min; standardized toothbrush and non-fluoridated toothpaste.<br>G2: 5 mL rinse (0.03% triclosan, 0.05% NaF, 5% xylitol), 1 min, twice daily for 15 days (under parental supervision); no food/drink for 30 min; standardized toothbrush and non-fluoridated toothpaste.	*Mutans streptococci* levels in dental plaque were evaluated using the Dentocult SM Strip Mutans test	15 days	After 15 days, 0.2% chlorhexidine gluconate was more effective than the triclosan–sodium fluoride–xylitol mouth rinse in reducing *S. mutans* in dental plaque, confirming its superior substantivity and bactericidal action.
Karjalainen et al., 1994 (Finland)	206<br>7-8 years	G1: Toothpaste containing 0.15% sodium monofluorophosphate<br>G2: Neutral sodium fluoride mouthrinse (0.2%)	Both groups received oral hygiene instruction and reinforcement. Test: 75 mL toothpaste every 2 months, used twice daily (unsupervised). Control: biweekly supervised mouthrinses + supervised brushing. Both: annual dental visits, individual instruction, dietary counseling, and fluoride varnish as needed.	Clinical exams using WHO criteria for dentin caries; blinded examiner; outcomes: DMFT and ADMFT (annual increment).	3 years	Stopping supervised school fluoride mouthrinses increases caries progression, reduces caries-free children, and raises restorative needs; thus, regular supervised use should be maintained or replaced only with

						equally effective preventive strategies.
Driscoll et al., 1992 (United States)	640<br>5-6 years	G1: Neutral NaF mouthwash at 0.2% (0.09% F).<br>G2: Neutral NaF tablet 2.2 mg (1.0 mg fluoride).<br>G3: Neutral NaF mouthwash at 0.2% (0.09% F) + Neutral NaF tablet 2.2 mg (1.0 mg fluoride).	G1: 0.2% NaF rinse (0.09% fluoride), 10 mL for 60 s (5 mL for kindergarten), once weekly at school under teacher supervision.<br>G2: daily NaF tablet (2.2 mg NaF ≈ 1 mg fluoride); chewed 20 s + 30 s swish then swallowed (first 5 years), later dissolved slowly in the mouth.<br>G3: combined regimen (weekly rinse + daily tablet).	DMFS	8 years	After 8 years, the combined regimen (rinse + tablet) showed the lowest caries increment, significantly lower than rinse alone; tablets also reduced caries without significant differences, supporting added benefit, though tablets were recommended for new programs due to lower cost and complexity.
Clark et al., 1985 (Canada)	425<br>6-7 years	G1: 0.2% sodium fluoride (NaF) mouthrinse.<br>G2: Topical application of water (placebo).	G1: weekly 10 mL 0.2% NaF rinse for 60 s under supervision (teachers/dental hygienists).<br>G2: semiannual prophylaxis (non-fluoridated paste + water application).<br>Both: routine school oral health education; no additional fluoride exposure.	DMFS and dfs Examination method: Visual and tactile exam according to standardized criteria.	20 months	Weekly 0.2% NaF mouthrinse significantly reduced caries in non-fluoridated settings (−34.2% DMFS permanent; −57.5% dfs primary), confirming a simple, effective, low-cost preventive strategy.

Abbreviations: ADMFT (approximal decayed, missing, and filled permanent teeth), CFU (colony-forming units), CFU/mL (colony-forming units per milliliter), CHX (chlorhexidine), dfs (decayed and filled primary tooth surfaces), dmf (decayed, missing, and filled primary teeth), DMFS (decayed, missing, and filled permanent tooth surfaces), DMFT (decayed, missing, and filled permanent teeth), F-MR (fluoride mouth rinse), MALDI-TOF (matrix-assisted laser desorption/ionization time-of-flight), MRS agar (De Man, Rogosa and Sharpe agar), MS (Streptococcus mutans), MSB agar (Mitis Salivarius Bacitracin agar), NaF (sodium fluoride), NDM (non-dialyzable material), PB (probiotic), PI (plaque index), ppm (parts per million), WHO (World Health Organization), w/v (weight per volume).

Table 3. General characteristics of the included studies evaluating the effects of fluoride varnish on dental caries management.

Author/ year (Country)	Population characteristics (N, Age, Gender)	Interventions	Protocol	Assessment Method	Follow-up	Main results
Narayan et al., 2017 (Imdia)	60<br>3-6 years	G1: Chlorhexidine varnish<br>G2: 0.2 ml of 10% povidone-iodo<br>G3: Fluor Protector<br>G4: Varnish placebo.	G1: CHX varnish, two applications 1 week apart.<br>G2: 0.2 mL 10% propidium iodide for 10 s, two applications 1 week apart.<br>G3: fluoride varnish, two applications 1 week apart.<br>G4: placebo varnish, same protocol.	CFU/mL	0, 30, 60 and 90 days	The fluoride varnish was the most effective in reducing *Streptococcus mutans*, outperforming chlorhexidine and povidone-iodine. All agents were more effective than the placebo, and the authors recommend future studies with larger samples and longer follow-up periods.
Duangthip et *al*., 2018 (China)	371<br>3-4 years	G1: 30% SDF - applied once a year.<br><br>G2: 30% SDF applied three times at weekly intervals at baseline.<br><br>G3: 5% NaF varnish applied three times at weekly intervals at baseline.	Standardized protocol: supine position; debris removal and tooth isolation; saliva control (no air drying); solution applied with microbrush for 10 s; no eating/drinking/rinsing for 30 min.	ICDAS criteria (codes 3–6) were used to assess lesion activity and progression<br><br>Indices used: dmfs and VPI (Visible Plaque Index)	0, 6, 12, 18, 24, and 30 months	5% NaF varnish (3 weekly applications) showed moderate caries arrest (34% cavitated lesions at 30 months), lower than annual SDF but similar to weekly SDF; comparable efficacy for ICDAS 3–4 (~51%), with less staining and no adverse effects..
Latifi-Xhemajle et al., 2019 (Canada)	427<br>6-30 months	G1: topical fluoride varnish applied four times per year (every three months) for two years<br>G2: no varnish application.	Children were examined and treated in preschool settings. Before each application, teeth were cleaned and dried with gauze. The varnish was applied to all tooth surfaces with a brush. Parents were advised not to allow eating or drinking for at least one hour after application.	ICDAS (codes 0–6). dmfs index and lesion prevalence by ICDAS stage.	0 and 24 months	Quarterly NaF varnish reduced caries by 49% (69.4% caries-free vs. 42.6% control), with lower ICDAS 01–06 lesions, confirming effective reduction in ECC incidence and severity in high-risk children.
Mabangkhru et al., 2020 (Thailand)	302<br>1-3 years	G1: 5% Sodium Fluoride varnish<br>G2: 38% Silver Diamine Fluoride	NaF 5% varnish and SDF 38% applied without caries removal; teeth cleaned and isolated; applied with microbrush (about 10 s for SDF; varnish covering lesion); no food/drink for 30 min; applications every 6 months.	Clinical examinations: Visual-tactile examination using WHO CPI prob. Indices: dmft, dmfs, and Visible Plaque Index (VPI).	0, 6 and 12 months.	6-monthly 5% NaF varnish showed limited dentin caries arrest, significantly less effective than 38% SDF; safe, well accepted, and non-staining.

Lam et al., 2021 (China)	323<br>3-4 years	G1: 5% sodium fluoride varnish<br>G2: Glass ionomer sealant	G1: 5% NaF varnish (Duraphat®), applied with microbrush to all teeth; no food/drink for 30 min; repeated every 3 months.<br>G2: GIC sealant (GC Fuji VII) using finger-press ART technique (cleaning, conditioning 10–15 s, drying, placement, adaptation with Vaseline-coated finger).	Clinical evaluation retention of the sealant; Indices: dmft, dmfs, and Visible Plaque Index (VPI), ceo-d e ICDAS.	0, 6 and 12 months	Quarterly application of fluoride varnish (NaFV) and single placement of glass ionomer sealant (GIS) showed similar effectiveness in preventing occlusal caries development in preschool children.
Phonghanyudh et et al., 2022 (Thailand)	290<br>1-3 years	G1: 38% Silver Diamine Fluoride (SDF)<br>G2: 5% Sodium Fluoride Varnish (NaFV)	Both groups: teeth cleaned/isolated (no caries removal).<br>G1: 38% SDF applied with microapplicator, rubbed for 10 s.<br>G2: 5% NaF varnish applied with microapplicator.<br>Both: no food/drink/rinse for 30 min; repeated every 6 months.	Indices: dmft, dmfs, Visible Plaque Index (VPI) e ICDAS.	0, 6, 12 and 18 months	After 18 months of follow-up, no statistically significant difference was observed in the ability to halt the progression of enamel caries in primary teeth between semiannual applications of 5% NaF varnish and 38% SDF in preschool children.
Sousa et al., 2022 (Brazil)	213<br>3-4 years	G1: Neutral fluoride gel (2% sodium fluoride)<br>G2: Fluoride varnish (sodium fluoride 5%)	G1: Neutral fluoride gel (pea-sized), brushed for 1 min; excess removed with gauze; repeated every 4 months for 1 year.<br>G2: Fluoride varnish (pea-sized), applied with microbrush after isolation/drying; no brushing on application day; repeated every 4 months for 1 year.	Indices: dmfs, visible plaque index (VPI), gingival bleeding (GB) e ICDAS.	0 and 12 months	After 12 months, 5% fluoride varnish showed similar effectiveness to 2% neutral fluoride gel in managing early childhood caries, resulting in a reduction in caries lesion progression. Therefore, both the patient and the dentist can choose which product to use, and public health services could benefit from the availability of a more affordable option.
Agarwal et al., 2022 (India)	515<br>3-4 years	G1: fluoride varnish *Fluoritop SR®* (22,600 ppm F)<br>G2: Water	G1: Fluoride varnish (0.3–0.5 mL) applied to all tooth surfaces; no liquids for 30 min and no solids for the rest of the day; at baseline, 6, and 12 months.<br>G2: Water applied using the same protocol (blinding)	Clinical examinations performed by trained and blinded dentists using ICDAS II. Questionnaires completed by parents.	0, 6 and 12 months	Fluoride varnish is safe and effective for preventing ECC in high-fluoride areas, providing additional benefit in high-risk children despite existing fluoride exposure.

Zheng et al., 2023 (China)	434 3-4 years	G1: 5% sodium FV G2:38% SDF	Both protocols: fluoride applied to all surfaces of the six upper anterior teeth using a microbrush; care to avoid gingival contact; no eating/drinking for 30 min.	Indices: dmfs and dmft (WHO, 2013), VPI	0, 1 day and 12 months.	At 12 months, 5% NaF varnish showed similar efficacy to 38% SDF (no difference in new caries or incidence), with no adverse effects, confirming it as a safe and well-accepted option.
Sirivichayakul et al., 2023 (Thailand)	155 4-6 years	G1: placebo control; (water); G2: 5% NaF varnish. G3: 38% SDF	G1: Placebo applied with microapplicator after cleaning (gauze/floss); focus on proximal surfaces; no food/drink for 30 min; reapplication every 6 months. G2: 0.25 mL NaF varnish applied with microapplicator after cleaning; focus on proximal surfaces; no food/drink for 30 min; reapplication every 6 months. G3: SDF applied with same protocol (≤1 drop, 25 µL per child); reapplication every 6 months.	Clinical examination, dmft, dmfs, Simplified Oral Hygiene Index (OHI-S), Interproximal Radiographs.	0, 6,12 and 18 months	After 18 months, semiannual 5% NaF varnish or 38% SDF showed no significant benefit over placebo for approximal caries prevention; lesion type/severity were key factors, with NaF showing a non-significant trend toward arresting non-cavitated lesions
Sorazabal et al., 2023	180 4-11 years	G1: 5% sodium fluoride varnish G2: Acidulated phosphate fluoride (APF) gel pH 3.5 (12,300 ppm) G3: Acidulated phosphate fluoride (APF) gel, pH 3.5 (12,300 ppm), applied with a toothbrush	G1: 5% NaF varnish (0.25–0.40 mL, pH 7), applied to all tooth surfaces twice yearly; no brushing/flossing or hard/sticky foods for 12 h. G2: 1.23% APF gel (tray, pH 3.5), applied for 1 min twice yearly; no rinsing/eating/drinking for 30 min. G3: 1.23% APF gel (toothbrush), applied for 2 min twice yearly; no rinsing/eating/drinking for 30 min	ICDAS II criteria.	0, 12 and 24 months	After 24 months, NaF varnish showed lower effectiveness than APF gel applied with a toothbrush, possibly due to reduced penetration into fissures and interproximal areas.
Manchanda et al., 2023 (China)	135 3-4 years	G1: 5% NaF varnish (Duraphat, Colgate-Palmolive, UK) G2: 5% NaF + tricalcium phosphate (TCP) varnish (Clinpro White Varnish, 3M ESPE, USA) G3: 5% NaF + casein phosphopeptide–amorphous calcium phosphate (CPP-ACP) varnish (MI Varnish,	G1–G3: Applied to all erupted primary teeth (≈0.25 mL) with microbrush after plaque removal and drying; same protocol across groups. Post-application: no food/drink for 2–4 h, no brushing, avoid hard/abrasive foods for 24 h; at follow-ups, toothbrush + 600 ppm fluoride toothpaste provided.	quantitative Real-Time Polymerase Chain Reaction (qRT-PCR)	0 and 24 months	Over 24 months, 5% NaF varnish showed similar antibacterial effects to Ca/P-added varnishes (no additional benefit), with no adverse effects, confirming safety and effectiveness.

		GC Corporation, Japan)				
Manchanda et al., 2024 (China)	570<br>3-4 years	G1: 5% NaF varnish (Duraphat® Varnish, Colgate-Palmolive Ltd, (UK)<br>G2: 5% NaF with TCP (Clinpro™ White Varnish; 3 M ESPE, St Paul, MN, USA)<br>G3: 5% NaF varnish with CPP-ACP (MI Varnish™; GC corporation, Tokyo, Japan)	All groups: teeth cleaned and isolated; 0.25 mL varnish applied with microbrush to all surfaces; no food/drink for 2–4 h and no brushing for 24 h; repeated quarterly over 24 months (3–6 applications).	Modified ICDAS-II (codes 2–9); Visible Plaque Index (VPI) for oral hygiene; microbial counts (qPCR for S. mutans and L. fermentum).	0 and 24 months	5% NaF varnish (Duraphat®) was as effective as Ca/P-modified varnishes (Clinpro™, MI Varnish™) in preventing and controlling caries in high-risk preschool children, confirming its established effectiveness.
Lam et al., 2024 (China)	479<br>3-4 years	G1: 5 % NaFV (Colgate Duraphat® varnish, Colgate-Palmolive (UK) Ltd.)<br>G2: glass-ionomer sealant (Fuji VII pink, GC Asia)	G1: 0.25 mL 5% NaF varnish (Duraphat®) applied to all teeth; no eating/drinking/rinsing for 30 min; reapplied every 3 months.<br>G2: GIC sealant (Fuji VII) using finger-press technique (cleaning, isolation, 20% polyacrylic acid conditioning, placement, petroleum jelly); no eating/drinking for 30 min.	Indices: DMFT, DMFS, and Visible Plaque Index (VPI), ceo-d e ICDAS.	0, 6, 12 and 18–24 months	No significant difference between NaF varnish and GIC sealant in preventing dentinal caries (≈17% each; p>0.05); similar effectiveness, with choice depending on practicality and child cooperation.
Khairy et al., 2025 (Egypt)	66<br>3-6 years	G1: Curodont Repair Fluoride Plus (VARDIS PROFESSIONAL, Switzerland) contains P11-4 peptide with 0.05% NaF.<br>G2: MI Varnish (GC Corporation, Japan) contains 2% CPP-ACP (casein phosphopeptide–amorphous calcium phosphate) with 5% NaF in varnish form.<br>G3: 5% sodium fluoride (NaF) in varnish form (Duraphat Varnish, Colgate, USA)	G1: CRFP applied after NaOCl (2%) and phosphoric acid (35%, 20 s) pretreatment; left for 5 min; reapplications at 2 and 4 weeks; no food/drink/rinse for 30 min.<br>G2: MI Varnish applied after isolation/drying; reapplications at 2 and 4 weeks; no food/drink/rinse for up to 4 h.<br>G3: Duraphat varnish applied as G2; same reapplication schedule and instructions.	Indices: dmft (WHO), PI (modified Silness and Löe plaque index), CAMBRA (Caries Management By Risk Assessment), ICDAS.<br><br>Laser fluorescence readings: DIAGNOdent Classic.	0, 3, 6, 9 and 12 mont hs	At 12 months, 5% NaF varnish (Duraphat) showed similar outcomes to MI Varnish in reducing lesion progression, though MI Varnish achieved deeper remineralization; CRFP showed superior caries arrest and the greatest remineralization depth with improved aesthetics.
Zeng et al., 2025 (China)	236<br>7-8 years	G1: 5% NaF varnish (Clinpro White Varnish, 3M ESPE)<br>G2: Oral health education<br>G3: 5% NaF Varnish (Clinpro White Varnish, 3M ESPE) + Oral health education	G1: 5% NaF varnish applied semiannually for 12 months (no further reinforcement).<br>G2: Oral health education only (leaflets), reinforced at 6 and 12 months.<br>G3: Combined varnish + education for 12 months; follow-up to 24 months.	DMFT/dmft, DMFS/dmfs, and DFS; ICDAS; DI-S; supragingival plaque analyzed via 16S rRNA sequencing	0, 60 12 and 24 months	Fluoride varnish alone showed limited effectiveness, being less effective than oral health education (especially in permanent molars); combined varnish + education was more effective, supporting integrated strategies for sustainable prevention.

Abbreviations: APF (acidulated phosphate fluoride), CAMBRA (Caries Management by Risk Assessment), CFU (colony-forming units), CPI (Community Periodontal Index), CPP-ACP (casein phosphopeptide–amorphous calcium phosphate), CRFP (Curodont Repair Fluoride Plus), DFS (decayed and filled surfaces), DI-S (Debris Index-Simplified), dmfs (decayed, missing, and filled primary tooth surfaces), dmft (decayed, missing, and filled primary teeth), FV (fluoride varnish), GB (gingival bleeding), GIS (glass ionomer sealant), ICDAS (International Caries Detection and Assessment System), ICDAS-II (International Caries Detection and Assessment System, second version), NaF (sodium fluoride), NaFV (sodium fluoride varnish), OHI-S (Simplified Oral Hygiene Index), PCoA (principal coordinates analysis), PI (plaque index), PSMs (primary second molars), qRT-PCR (quantitative real-time polymerase chain reaction), rRNA (ribosomal ribonucleic acid), SDF (silver diamine fluoride), TCP (tricalcium phosphate), VPI (visible plaque index), WHO (World Health Organization).

Table 4. General characteristics of the included studies evaluating the effects of silver diamine fluoride on dental caries management.

Author/ year (Country)	Population (N, Age, Gender)	Interventions	Protocol	Assessment Method	Follow-up	Main results
Ammar et al., 2022 (Alexandria, Egypt)	50 children, 4–6 years old	G1: NSF<br>G2: 38% SDF	G1/G2: single SDF application; debris removal, partial isolation; lesions dried (5 s); one drop applied with microbrush for 1 min; excess removed; no eating/drinking for 1 h	DMFT, ICDAS (Code 5 for inclusion), *Streptococcus mutans (S. mutans)* and *lactoba cilli* counts as CFU/mL	1 month	NSF showed a significantly greater percentage reduction in *S. mutans* count (21.28%) than SDF (10.46%) (p=0.002). Both had a high rate of lesion interruption.
Cleary et al., 2022 (Michigan, USA)	98 children, 2 to 10 years	G1: Silver Diamine Fluoride (SDF) 38% (Advantage Arrest Silver Diamine Fluoride 38%, Elevate Oral Care USA)<br>G2: Restorative Treatment (RT) [(Not specified)]	G1: 38% SDF applied semiannually after cleaning, isolation, and drying; excess blotted.<br>G2: Restorative treatment (RT) per guidelines, with anesthesia/behavior guidance as needed.	ICDAS (International Caries Detection and Assessment System), DMFT (Caries experience), Wong-Baker Scale (Child's perception of appearance, feeling, visit), Periapical and Interproximal radiographs	12 months	Lesions treated with SDF had significantly more failures (minor and major) at 12 months compared with RT. Providers considered SDF faster (5 min vs. 30 min).

Duangthip et al., 2018 (Hong Kong, China)	888 children (M: 519 F:369) 3-4 years	G1: 12% SDF (Cariestop Biodinâmica) anual; G2: 12% SDF (Cariestop Biodinâmica) semianual; G3: 38% SDF (Saforide; Toyo Seiyaku) anual; G4: 38% SDF semianual (Saforide; Toyo Seiyaku Kasei Co. Ltd.)	G1/G2: 12% SDF (Cariestop) and 38% SDF (Saforide), applied annually or semiannually. Food debris and plaque removed; isolation with gauze. SDF applied with a micro-applicator for approximately 1 minute. Excess removed with gauze. Instruction: Refrain from eating, drinking, or rinsing for 30 minutes.	dmft (Caries experience), Parental Questionnaires (Demographics, diet, oral hygiene, pain reports). Wong-Baker Scale (Child's perception of appearance, feeling, visit).	30 months	Darkening of the lesions was common in all groups (36.7% to 76.3%). There were no acute systemic diseases. Minor adverse effects (pain/swelling of the gums, whitening) were uncommon and did not differ significantly between the groups.
ElGhandour et al., 2021 (Alexandria, Egypt)	100 children, 2-5 years	G1: SDF 38% (Advantage Arrest Silver Diamine Fluoride 38%, Elevate Oral Care USA), G2: ART-GC Fuji IX, GC America	G1: 38% SDF applied biannually after debris removal and drying; applied with microbrush, left 1 min, then covered with petroleum jelly. G2: ART with high-viscosity GIC (Fuji IX) after peripheral caries removal; isolation; finger-press technique with petroleum jelly; excess removed.	ICDAS II scoring. dmft score. OHRQoL Scores: P-CPQ, FIS	12 months (quarterly follow-ups)	SDF demonstrated greater success in halting caries than ART at 12 months (94.4% SDF vs. 34.3% ART). Both treatments significantly improved OHRQoL, with no statistically significant difference between them.
Gao et al., 2020 (China)	1070 children, 3–4 years	G1: received semi-annual applications of 25 % silver nitrate +5 % NaF varnish G2: semi-annual applications of 38 % SDF solution + placebo varnish.	G1/G2: Treatments applied only to carious lesions with microbrush (~5 s per lesion); no eating/drinking for 30 min; applications at baseline and follow-ups (6, 12, 18, 24, 30 months).	DMFS, VPI, Parental Questionnaire	30 months	The efficacy of $AgNO_3$+NaF was comparable (non-inferior) to that of 38% SDF in interrupting ECC. Lesions on anterior teeth and buccal surfaces were more likely to be interrupted.
Jiang et al., 2020 (Hong Kong, China)	194 children, 3–4 years	G1: SDF 38% (Saforide, Toyo Seiyaku Kasei Co., Osaka, Japan) + ART (Ketac-molar, 3 M ESPE,	G1: 38% SDF (Saforide) applied pre-ART without caries removal (10 s, microapplicator); isolation; no	DMFS	24 months (evaluations every 6 months)	Prior application of SDF did not significantly affect the success rate of ART restorations ($p > 0.05$). It was faster to place

		Germany), G2: Placebo (tonic water) + ART (Ketac-molar, 3 M ESPE)	food/drink for ≥30 min; ART (GIC) placed after 10 weeks.			ART restorations in lesions treated with SDF (4.8 min vs. 5.1 min).
Jiang et al., 2022 (Hong Kong, China)	194 children, child under age six	G1: SDF 38% (Saforide, Toyo Seiyaku Kasei Co., Osaka, Japan) + ART restorations, Germany), G2: Placebo (tonic water) + ART	G1: 38% SDF (Saforide) applied pre-ART without caries removal (10 s, microapplicator); isolation; no eating/drinking for ≥30 min; ART (GIC) placed after 10 weeks and covered with Vaseline.	DMFT score, ECOHIS, CIS, FIS	24 months (evaluations every 6 months)	Parental satisfaction with dental health improved significantly in both groups. At 24 months, the control group (Placebo/ART) had a significant worsening of OHRQoL (higher total CIS and ECOHIS scores), which did not occur in the SDF group.
Mohammed et al., 2022 (Egypt)	30 children, 3–6 years	G1: High Viscosity Glass Ionomer Cement (HVGIC) (GIC Fuji™ II capsule, GC, USA) + G2: SMART- SDF 38% (Kids-e-Dental Company) + HVGIC (GIC Fuji™ II capsule, GC, USA)	G1: GIC (Fuji II) restoration per manufacturer's instructions.<br>G2 (SMART): 38% SDF applied (≥1 min, microbrush) after selective dentin removal; excess removed and dried (15 s); GIC (Fuji II) placed after 1 week.	Clinical Success/Failure of the restoration	6 and 12 months.	SMART had higher success rates (60% in 12M) than ART (53.33% in 12M). The difference was not statistically significant.performance was clinically acceptable.
Phonghanyud et al., 2022 (Thailand)	290 children, 1–3 years	G1: 38% SDF (Topamine, DentaLife, Australia), G2: Sodium Fluoride (NaF) Varnish 5% (Duraphat, Colgate-Palmolive, Guildford, Surrey, UK)	G1/G2: Teeth cleaned/isolated (no caries removal); G1: 38% SDF applied/rubbed for 10 s; G2: 5% NaF varnish applied; no eating/drinking/rinsing for 30 min; repeated every 6 months.	ICDAS criteria (Code 2 or 3 for inclusion), dmft/dmfs, VPI	18 months	SDF and NaF varnish had comparable efficacy in halting enamel caries (59.1% SDF vs. 58.8% NaF; p=0.873).
Quritum et al., 2024 (Alexandria, Egypt)	360 children, ≤ 4 years	G1: Nano Silver Fluoride (NSF) (Laboratory prepared (AgNPs PEGylated + NaF in deionized water), G2: 38% SDF (Advantage	G1/G2: No caries removal; teeth cleaned and isolated; no eating/drinking for 30 min.<br>SDF group: one drop applied with microbrush (excess	dmft, A-ECOHIS, CIS, FIS, PI, Sugar Consumption Score (0-8).	6 months	Both agents significantly improved OHRQoL. NSF had a significantly better impact on OHRQoL than SDF (B = -5.02, p = 0.001), possibly

		Arrest, Elevate Oral Care LLC., FL, USA)	removed), left to dry for 1 min.			due to the absence of staining.
Ruff et al., 2023 (New York, EUA)	2998 children, M:1432 F: 1566 5 to 13 years, mean age 6.6 (1.2) years	G1: SDF 38% (Not specified) + Fluoride Varnish, G2: ART (Glass ionomer-Not specified) + Fluoride Varnish	G1: Biannual SDF applied to cavitated lesions and pits/fissures (≥30 s), air-dried ≥60 s, followed by fluoride varnish.<br>G2: GIC sealant after cavity conditioner (10 s); mixed capsule (10 s, 4000 rpm) and applied using finger-sweep technique.	ICDAS (Code 5 or 6 for untreated dental caries), Parental Demographic Data	2 years	The interruption and prevention rates of SDF were non-inferior to those of the active control (ART/GIC). There were no adverse events.
Sharawat et al., 2025 (India)	150 children, 4–9 years old, mean age 5.84 (1.45) years	G1: Single-visit SMART- 38% SDF (FAgamin Silver Diamine Fluoride) + GIC (3M ESPE Ketac cement, Germany); G2: Two-visit SMART- 38% SDF, Tedequim, Argentina) + GIC	G1/G2 (SMART) SDF 38% FAgamin. Site Preparation: Selective caries removal to firm dentin. Lip protection (Vaseline). Application: SDF applied and rubbed with micro-brush for 1 min, left undisturbed for 1 min. Conditioning: Dentin Conditioner (10% Polyacrylic Acid) was applied before GIC placement.	DMFT, Innes criteria (for restoration assessment).	12 months (6 and 12 months).	There was no significant difference in success rates at 12 months (68.0% single visit vs. 82.7% two visits; p=0.051). Major failures (irreversible pulpitis) occurred only in the single visit group (4.0%).
Sun et al., 2024 (Hong Kong, China)	298 children, 3–4 years	G1: SDF 38% Immediate Rinsing (Advantage Arrest, Elevate Oral Care, FL, USA); G2: SDF 38% No Rinsing 30 min (Advantage Arrest, Elevate Oral Care, FL, USA)	G1/G2 (SDF 38% Advantage Arrest), annual application. Site Preparation: Visible plaque and food debris removed, isolation. Application: SDF applied with microbrush for 60 seconds. Post-Operative: G1 (Immediate Rinsing): Instructed to rinse immediately. G2 (No Rinsing): Instructed	DMFT/DMFS (Caries experience), VPI (Visible Plaque Index), ICDAS (Code 5 or 6 for dentin caries)	6 months	Not rinsing for 30 minutes was not superior to immediate rinsing in stopping caries (61% G B vs. 65% G A; p=0.28).

			not to rinse, drink, or eat for 30 min.			
Vollú et al., 2019 (Rio de Janeiro, Brazil)	67 children, 2–5 years	G1: SDF 30% (Cariostop, Biodinâmica, Paraná, Brazil), G2: ART (Ketac Molar Easy Mix, 3M ESPE)	G1: 30% SDF (Cariostop) applied biannually after cleaning, protection, isolation/drying; applied with microbrush for 3 min; excess removed and rinsed. G2: ART with GIC (cleaning, isolation, selective caries removal, conditioning, placement/adaptation, digital pressure, occlusal adjustment, protection); no eating for 1 h	DMF-T, ICDAS (Scores 5 or 6), OHRQoL: B-ECOHIS (Brazilian version), CIS, FIS, Facial Image Scale (5 categories: very happy to very unhappy).	12 months (3, 6 and 12 months)	Treatment time with SDF was significantly shorter (Mean 6.97 min) than with ART (Mean 13.88 min) ($p<0.001$). ART had less impact on the OHRQoL, but only on the parental distress subscale ($p=0.012$).
Yassin et al., 2023 (Alexandria, Egypt)	165 children, 15-48 months	G1: 38% SDF (Advantage Arrest, Elevate Oral Care, FL, USA); G2: NaF 5% varnish (Alpha-Pro® White Varnish, USA)	G1/G2: single application. Cleaning and partial isolation. Lip/skin protection with Vaseline. SDF applied with disposable microbrush, "painted" for 10 s. Excess removed and solution left to dry for 1 minute. Do not eat or drink for 30 min.	DMFS, ICDAS, Plaque Index, Sugar Consumption Score (0-8).	6 months	The overall interruption rate did not differ significantly ($P=0.08$). SDF was significantly more effective in advanced lesions (ICDAS 5/6) (60.3% vs. 50.0%; $P=0.01$). NaF/MI may be a non-staining alternative.
Zheng et al., 2023 (Hong Kong, China)	688 children, 3–4 years	G1: 38% SDF, G2: 5% NaF	G1/G2: 38% SDF or 5% NaF varnish applied by independent operator; one application per surface of the six upper anterior teeth using microbrush; care to avoid gingival contact.	DMFS, VPI	12 months	SDF was not superior to FV for caries prevention. Child cooperation (71% vs. 70%) and parental satisfaction (71% vs. 69% satisfied) were similarly high.

Abbreviations: ART (Atraumatic Restorative Treatment), DMFS (Decayed, Missing, and Filled Surfaces), DMFT (Decayed, Missing, and Filled Teeth), ECOHIS (Early Childhood Oral Health Impact Scale), FIS (Family Impact Scale), NaF (sodium

fluoride), NSF (Nano Silver Fluoride), OHRQoL (Oral Health-related Quality of Life), P-CPQ (Parental-Caregiver Perceptions Questionnaire), PI (Plaque Index), RT (Restorative Treatment), SDF (Silver Diamine Fluoride), VPI (Visible Plaque Index).

Table 5. General characteristics of the included studies evaluating the effects of glass ionomer cement on dental caries management.

Author/ year (Country)	Population (N, Age, Gender)	Interventions	Protocol	Assessment Method	Follow-up	Main results
Aly et al. (2023) (Cairo, Egypt)	67 children (34 SMART, 33 ART). 5–9 years old (mean 6.01 ± 1.15 years). M: 36, F: 31	G1: SMART-SDF 38% + GIC capsule (Fuji IX GP® EXTRA) G2: ART (GIC capsule Fuji IX GP® EXTRA)	G1 (SMART): Isolation/protection; debris removal without caries excavation (except margins); cavity dried; SDF applied with microbrush, air-dried 1 min, excess blotted. G2: ART per Frencken protocol.	Modified USPHS Criteria (clinical performance evaluation), pre-and post-operative photos, Cost-effectiveness analysis (micro-cost approach, labor, material), FBRS.	6 and 12 months	Both techniques showed comparable clinical performance and survival (SMART: 11.8 months; ART: 11.6 months). Mean treatment time for SMART was significantly shorter (7.8 min.) than for ART (15 min). SMART showed a statistically significant lower mean total cost per restoration.
Bansal et al. (2023) (New Delhi, India)	226 children, 4–8 years old (mean 5.8 ± 1.28 years), M: 129, F: 97	G1: SMART:38% SDF (Fagamin®, Tedequim SRL, Bv.de los Polacos, Cordoba, Argentina) + GIC (GIC Ketac™ Molar (Deutschland), G2: ART (GIC Ketac™ Molar)	G1 (SMART): Minimal caries removal; 38% SDF applied for 1 min, excess absorbed, rinsed/dried, followed by GIC restoration. G2: Conventional drill-and-fill with selective excavation (bur) followed by GIC restoration.	ICDAS (scores 5 or 6 for inclusion), Intraoral periapical X-rays, SEM, FIS	Every 6 months, up to 24 months	No significant difference in GIC restoration success rates between SMART (38.4%) and conventional (45.8%) techniques at 24 months ($p = 0.105$). The SMART technique was better accepted by children (79%) compared to the conventional technique (56%) ($p < 0.001$).

Bodur et al., 2025 (Türkiye)	86 children. 5–9 years old (mean 7.15 ± 1.14 years), M: 44 boys, F: 42 girls	G1. ART- Equia Forte HT G2. ART- Fuji II LC G3.ART- Cention N G4- ART- ChemFil Rock.	G1: Capsule-mixed (10 s), single-step placement; light-cured 20 s; Equia Forte Coat applied and light-cured 20 s. G2: Capsule-mixed (10 s), single-step placement; light-cured 20 s. G3: Hand-mixed (1:1, 45–60 s); applied and self-cured ~4.5 min. G4: Capsule-mixed (12 s), single-step placement; working time 90 s + self-curing ~4.5 min.	Modified USPHS Criteria (retention, marginal discoloration, marginal adaptation, color stability, anatomical form). ICDAS II (scores 5 and 6) for inclusion, Periapical radiographs, digital photographs	12 months	Fuji II LC was the most successful in terms of retention (lowest failure rate of 7.14%) ($p < 0.05$). Cention N had the lowest retention success rate (25% failure). Equia Forte HT had the highest success rate regarding color stability.
Cabral et al. (2018) (Brazil)	56 children, Age: 5–7 years (mean 7.06 ± 0.56 years), M: 22, F: 34	G1: Clinpro XT Varnish (CXT), G2: Fuji IX GP FAST (FJ) (GC Co.).	G1 (CXT): 37% phosphoric acid (15 s), rinse/dry; material placed in pits/fissures and light-cured 30 s. G2: GIC (Fuji IX GP Fast) after conditioning (15 s); capsule mixed (10 s) and applied using finger-press ART technique.	ICDAS II, Sealant retention rates (traditional method and modified categorization), Cariogram-based form	6, 12 and 24 months	FJ sealants (HVGIC) were retained longer compared to CXT sealants ($p < 0.05$). Both materials were equally effective in preventing cavitated dentine lesions over 24 months ($p = 0.99$).
Faustino-Silva et al. (2019) (Rio Grande do Sul, Brazil)	25 children, 18–36 months old	G1: Ketac Molar Easymix® (3M ESPE), G2: VitroMolar® (DFL)	ART procedure: relative isolation, no anesthesia; caries removal with hand instruments; 11.5% polyacrylic acid conditioning (10 s); rinse/dry; GIC placement with 30 s digital pressure; occlusal adjustment; surface protection (AlfaBond®).	ART Criteria (success codes 0, 1, 7; failure codes 2, 3, 4, 8). Modified USPHS criteria (4-year evaluation), VPI, GBI.	1, 2 and 4 years	ART was effective in managing ECC. The total ART success percentage was 82.9% after 4 years. No statistically significant difference was found in clinical performance between the two HVGICs ($p > 0.05$).

Garbim et al. (2024) (São Paulo, Brazil)	152 children, 4–8 years old	G1: Equia Forte (EF) (GC CORP) G2: Riva Self Cure (RSC) (SDI).	G1/G2: ART with selective caries removal; conditioning (10 s), rinse/dry; sectional matrix + wedge; GIC capsule mixed (10 s) and applied with finger-press; excess removal, contacts checked; matrix removed, flossing; protective coating light-cured (20 s).	Roeleveld criteria (scores 0-10 considered success for occlusoproximal), Measurement of Cavity Volume in mm³ (using WHO probe to measure dimensions), Session time recorded for cost calculation, Cost-effectiveness analysis using Monte-Carlo.	2, 6, 12, 18 and 24 months	No significant difference in survival rates (EF: 45%; RSC: 32%). RSC was more cost-effective (total cost USD 19.30 vs. EF USD 25.48) after 24 months ($p < 0.001$).
Hesse et al. (2021) (Amsterdam, Netherlands)	187 children, 6–8 years old	G1: ART Sealants: Fuji IX (GC Europe) and Maxxion R (FGM) G2: Control Group-Nonsealed teeth.	Hand-mixed GIC (1:1) sealant using ART protocol: cleaning, isolation, conditioning (20 s), rinse/dry; finger-press placement; excess removal and occlusal adjustment; petroleum jelly protection; no eating for 1 h.	ICDAS, Nyvad criteria and DMFT-score	3, 6, 12, 18, 24 and 36 months (3 years)	Fuji IX (45.4%) showed significantly higher retention than Maxxion R (25.4%) ($p < 0.001$). There was no statistical difference between sealed and nonsealed molars regarding the development of cavitated dentin lesions ($p = 0.70$).
Moura et al. (2019) (Piauí, Brazil)	243 children, 2–6 years old	G1: Ketac Molar® (3M / ESPE) G2: Vitro Molar® (DFL).	G1/G2: ART protocol with selective caries removal (hand instruments; opener if needed); conditioning with polyacrylic acid (10 s), rinse (5 s), and drying; cavities randomized to materials.	ART Criteria (scores 0 to 9, success/failure).	6 and 12 months	Restorations using HVGIC (Ketac Molar®) were more successful than those with low-cost GIC (Vitro Molar®) after 12 months (PR = 1.07).
Oliveira et al. (2021) (São Paulo, Brazil)	145 children, 3–10 years old	G1: Hand-Mixed-Fuji IX Gold Label® (GC Europe), G2: Encapsulated GIC (ENC)-Equia Fill® (GC Europe).	G1: Hand-mixed GIC placed with spatula; finger-press (10 s); occlusion checked after 3–5 min; petroleum jelly protection. G2: Capsule-mixed	Frencken and Holmgren Criteria (occlusal), Roeleveld Criteria (occlusoproximal), Periapical radiographs	24 months	No statistically significant difference in survival rate was found between Hand-Mixed (60.9%) and Encapsulated (59.3%) GIC after 24 months ($p = 0.626$).

			GIC applied with applicator; finger-press (10 s); occlusion checked after 3–5 min; petroleum jelly protection; matrices/wedges for occlusoproximal cavities.			
Pesaressi et al. 2023 (Lima, Peru)	187 children, aged 3–7 years (average 5.5)	G1: Encapsulated GIC-EQUIA Fil (GC Corporation)-ART Class II with proximal retention grooves, G2: EQUIA Fil (GC Corporation)-ART Class II without proximal retention grooves.	ART protocol: hand excavation; cavity size recorded; G1 included proximal retention; 20% polyacrylic acid conditioning (15 s); matrix + wedge; HVGIC capsule mixed and placed; finger-press (40 s); excess removal; occlusal adjustment after 5 min; surface coat light-cured; no eating for 1 h.	ART Criteria (survival, codes 1 and 2 success), photographic documentation (6 and 12 months), Visible Plaque Index and Gingival Bleeding Index.	6 and 12 months	Proximal retention grooves increased the survival rate of ART Class II restorations after 12 months. Success rates at 12 months: With grooves: 91.8%; Without grooves: 77.2%.
Rodrigues et al. (2025) (Rio de Janeiro, Brazil)	118 participants, 2–5 years old (mean 3.53 ± 1.03 years)	G1: SDF 30% (Cariestop, Biodynamics) G2: ART-GIC Ketac Molar Easy Mix 3M ESPE.	G1: 30% SDF applied for 3 min after cleaning/isolation; excess removed and rinsed; reapplied every 6 months. G2: ART with selective caries removal; conditioning (10 s), rinse/dry; GIC placement with finger-press (30 s); occlusal adjustment; petroleum jelly; no eating/drinking for ≥1 h.	Outcomes: ICDAS (5–6), DMF-t, Frankl Behavior Scale, FIS, caries activity (soft/hard dentin), radiographs, socioeconomic/demographic data, and parental aesthetic perception.	12 and 24 months	SDF was similar to ART in arresting dentin caries lesions. SDF demonstrated a significantly shorter treatment time (6.08 min) compared to ART (13.58 min).

Salas Huamani et al. (2019) (São Paulo, Brazil)	78 children, 6–8 years old (mean age 6.49–6.62 years)	G1: ART (Ketac Molar Easymix®; 3M ESPE, São Paulo, Brazil), G2: OHES + ART (Ketac Molar Easymix®; 3M ESPE, São Paulo, Brazil).	G1/G2: Only hand instruments were used for opening and cleaning the cavities in primary molars. Cleaned cavities were re stored with a high-viscosity glass-ionomer (Ketac Molar Easymix®; 3M ESPE, São Paulo, Brazil).	m-VPT-anxiety, m-VAS- anxiety subjective.	Measures before, during, and after treatment	The educational strategy (OHES) associated with ART showed a positive effect in modulating objective stress markers (Heart Rate and salivary alpha-amylase) during critical excavation. The ART-only group showed higher Heart Rate during "Deep excavation".
Schraverus et al. 2021 (Amsterdam, Netherlands)	77 children, 5–9 years old (mean 6.81 ± 0.87 years)	G1: MIH-affected molars that remained unsealed G2: MIH-affected molars that received GIC capsule (Fuji Triage Pink; GC Europe)	School-based GIC sealant: isolation; conditioning (20 s), rinse/dry; capsule mixed (10 s) and applied/spread into fissures; light-cured 30 s; occlusal adjustment; protective coat light-cured 20 s.	Ghanim criteria (for PEB). ICDAS (for dental caries). Sealant retention criteria (0/1 success, 2 failure).	6 and 12 months	GIC sealant was effective in preventing dentin caries lesions. GIC sealant application was not associated with the prevention of PEB in MIH-affected molars.
Silva et al. 2021 (São Paulo, Brazil)	108 children, 4-8 years old	G1: HVGIC: Fuji IX (GC Corporation), G2: CHC + HVGIC-Hydro C; Dentsply Sirona, USA + Fuji IX (GC Corporation)	G1: Selective caries removal; conditioning, rinse/dry; HVGIC (Fuji IX) restoration with finger-press; matrix for occlusoproximal cavities. G2: Same protocol with calcium hydroxide liner before HVGIC; occlusion checked; petroleum jelly protection.	Roeleveld and Frencken criteria (restoration survival), interproximal X-ray (bitewing), Oral Hygiene Index (Greene and Vermillion index)	6, 12 and 24 months	The HVGIC (alone) group showed a higher restoration survival rate (73.3%) compared to the CHC+HVGIC group (50%) after 2 years. Pulp vitality success rates were comparable.

de Medeiros Serpa et al. (2017) (Paraíba, Brazil)	86 patients aged 4 to 8 years of both genders	G1: Single-surface cavity restored with GIC (Ketac™ Molar Easy Mix). G2: Single-surface cavity restored with composite resin (Filtek™ Z250). G3: Two-surface cavity restored with GIC (Ketac™ Molar Easy Mix). G4: Two-surface cavity restored with composite resin (Filtek™ Z250).	G1/G3: ART without anesthesia; selective caries removal; conditioning, rinse/dry; matrix + wedge; hand-mixed GIC with finger-press; excess removal, occlusal adjustment, varnish; no chewing for 1 h. G2/G4: Selective caries removal; 10% phosphoric acid etch; adhesive (Prime & Bond); composite (Z250) incremental technique (40 s curing/layer); $Ca(OH)_2$ liner in deep cavities; occlusal adjustment.	Self-formulated clinical and radiographic criteria (Satisfactory/Unsatisfactory categories), Parent interviews	12 months	Restorations were clinically successful in 89.3% of cases after 12 months. There was no statistical difference between GIC and Composite Resin performance.

Abbreviations: ART (Atraumatic Restorative Treatment), CXT (Clinpro XT Varnish), DMFT (Decayed, Missing, and Filled Teeth index), ECC (Early Childhood Caries), EF (Equia Forte), FBRS (Frankl Behavior Rating Scale), FIS (Facial Image Scale), GBI (Gingival Bleeding Index), GIC (Glass Ionomer Cement), HVGIC (High-Viscosity Glass Ionomer Cement), MIH (Molar Incisor Hypomineralization), m-VAS (Modified Visual Analogue Scale), m-VPT (Modified Venham Picture Test), OHES (Oral Health Education Strategies), PEB (Post-eruptive Breakdown), RSC (Riva Self Cure), SDF (Silver Diamine Fluoride), SEM (Sound, Eye and Motor), SMART (Silver Modified Atraumatic Restorative Treatment), USPHS (United States Public Health Service), VPI (Visible Plaque Index), WHO (World Health Organization Probe).

Thank you for this valuable suggestion. The manuscript was revised according to the PRISMA recommendations for scoping reviews.
In accordance with the reviewer's suggestion and the PRISMA guidelines:

1. – Lines 2-4
2. – Lines: 17-46
3. – Lines: 50-83
4. – Lines: 114-118
5. – Lines: 107-111
6. – Lines: 137-158
7. - Lines: 159-178
8. – Lines: 163-165
9. – Lines 180-188
10. –Lines: 191-197
11. -Lines: 193-196
12. - Lines: Not applicable

13. - Lines: 198-210
14. – Lines: Figure 1
15. – Lines: Supplementary Tables 1,2,3 and 4
16. - Not applicable
17. – Lines: 430-471
18. – Lines: 431-463
19. – Lines: 524-536
20. – Lines: 549-550
